# CNFE-SE: a novel approach combining complex network-based feature engineering and stacked ensemble to predict the success of intrauterine insemination and ranking the features

**DOI:** 10.1186/s12911-020-01362-0

**Published:** 2021-01-02

**Authors:** Sima Ranjbari, Toktam Khatibi, Ahmad Vosough Dizaji, Hesamoddin Sajadi, Mehdi Totonchi, Firouzeh Ghaffari

**Affiliations:** 1 School of Industrial and Systems Engineering, Tarbiat Modares University, Tehran, Iran; 2 Department of Genetics At Reproductive Biomedicine Research Center, Royan Institute for Reproductive Biomedicine, ACECR, Tehran, Iran; 3 Department of Reproductive Imaging, Reproductive Biomedicine Research Center, Royan Institute for Reproductive Biomedicine, ACECR, Tehran, Iran; 4 Department of Andrology, Reproductive Biomedicine Research Center, Royan Institute for Reproductive Biomedicine, ACECR, Tehran, Iran; 5 Department of Endocrinology and Female Infertility, Reproductive Biomedicine Research Center, Royan Institute for Reproductive Biomedicine, ACECR, Tehran, Iran

**Keywords:** IUI outcome prediction, Complex networks, Feature engineering, Stacked ensemble classifier, Feature selection

## Abstract

**Background:**

Intrauterine Insemination (IUI) outcome prediction is a challenging issue which the assisted reproductive technology (ART) practitioners are dealing with. Predicting the success or failure of IUI based on the couples' features can assist the physicians to make the appropriate decision for suggesting IUI to the couples or not and/or continuing the treatment or not for them. Many previous studies have been focused on predicting the in vitro fertilization (IVF) and intracytoplasmic sperm injection (ICSI) outcome using machine learning algorithms. But, to the best of our knowledge, a few studies have been focused on predicting the outcome of IUI. The main aim of this study is to propose an automatic classification and feature scoring method to predict intrauterine insemination (IUI) outcome and ranking the most significant features.

**Methods:**

For this purpose, a novel approach combining complex network-based feature engineering and stacked ensemble (CNFE-SE) is proposed. Three complex networks are extracted considering the patients' data similarities. The feature engineering step is performed on the complex networks. The original feature set and/or the features engineered are fed to the proposed stacked ensemble to classify and predict IUI outcome for couples per IUI treatment cycle. Our study is a retrospective study of a 5-year couples' data undergoing IUI. Data is collected from Reproductive Biomedicine Research Center, Royan Institute describing 11,255 IUI treatment cycles for 8,360 couples. Our dataset includes the couples' demographic characteristics, historical data about the patients' diseases, the clinical diagnosis, the treatment plans and the prescribed drugs during the cycles, semen quality, laboratory tests and the clinical pregnancy outcome.

**Results:**

Experimental results show that the proposed method outperforms the compared methods with Area under receiver operating characteristics curve (AUC) of 0.84 ± 0.01, sensitivity of 0.79 ± 0.01, specificity of 0.91 ± 0.01, and accuracy of 0.85 ± 0.01 for the prediction of IUI outcome.

**Conclusions:**

The most important predictors for predicting IUI outcome are semen parameters (sperm motility and concentration) as well as female body mass index (BMI).

## Background

Infertility is defined as the failure of the female partner to conceive after at least one year of regular unprotected sexual intercourse [1]. More than 186 million people of the world's population specifically people living in developing countries are suffering from infertility [2]. In most cases, the causes of infertility are not clear, which complicates the treatment procedure. These problems have been exacerbated for several reasons, such as lifestyle changes, infection, and genetic issues. In many cases, the only way to get pregnant has been through the use of assisted reproductive technology (ART), and its performance has not yet been optimized [3].

Every year, more than 1.5 million ART cycles are carried out all over the world [4]. ART consists of three basic procedures including intrauterine insemination (IUI), in-vitro fertilization (IVF) and intracytoplasmic injection (ICSI) which are generally carried out in different steps of the treatment [5]. The first-line treatment, second and the third stages of ART are IUI, IVF, and ICSI, respectively [6]. In comparison with other sophisticated methods of ART, IUI has been considered as the easiest, minimally invasive and less expensive one. Most of the recent researches have shown the efficacy of IUI [6, 7].

IUI outcome prediction is a challenging issue which the ART practitioners are dealing with. Predicting the success or failure of IUI based on the couples' features can assist the physicians to make the appropriate decision for suggesting IUI to the couples or not and/or continuing the treatment or not for them [5].

Machine Learning approaches, as the modern scientific discipline, concentrates on how to detect the hidden patterns and extract the information from data. Machine learning provides different methods and algorithms to predict the output from some input predictors which can be used for clinical decision making [8].

To the best of our knowledge, many previous studies have been focused on predicting the IVF and ICSI outcome using machine learning methods as summarized in Table 1.

**Table 1:** Summarizing the previous studies of predicting ART outcome

Research problem	Dataset	Features	Analytical method	Remarks
Predicting IVF outcomes	5275 records	67 different features	Combination of Decision Tree and Genetic algorithm	Low predictive accuracy with 73%
Patient-specific predictions of outcome after IUI	1438 patients who underwent 3375 IUI cycles	8 features	Logistic regression analysis	A few numbers of features
Predictive modeling of implantation outcome in IVF	3898 embryos	18 features	Naive Bayes, Decision Tree, K Nearest Neighbors, SVM, multilayer perceptron, radial basis function network	A small number of features
Determine the impact of sperm morphology on the success of IUI	412 couples with 530 IUI cycles	12 features	statistical analysis	A few samples studied
Outcome prediction of IUI based on sperm morphology and progressively motile sperm count	4251 first IUI cycles of 1166 couples	9 features	multivariable logistic regression	A few features considered
Predicting live birth after IVF complete cycle	113,873 women data	Age and duration of infertility	Logistic regression	A few difference makers considered
Identifying and choosing the best sperms for ICSI	219 patients	13 features	Naive Bayes, SVM, MLP, IBK, K-Star, Random Committee, J48, Random Forest	Small set of patients
IVF outcome prediction relying on endometrial transcriptions	25 patients	20 feature	PCA and HCA clustering	Small number of patients
Predicting Implantation Outcome of IVF and ICSI	the data of 486 patients	21 features	SVM, Adaboost, RPART, RF, 1-NN	A few features considered
Predicting the impact of homologous semen on the success rate of IUI	556 couples with 1401 IUI cycles	16 features	Logistic regression	Small dataset
Assessing the effects of FSH and clomiphene citrate on infertile women with unexplained infertility	2259 IUI cycles of 684 couples	6 features	Logistic regression	A few determinative factors studied
Outcome prediction of ART	257 infertile couples	12 features	ANN	Small dataset
Prediction of implantation after blastocyst transfer in IVF or ICSI	1052 patients in	32 features	Random Forest, Multivariate logistic regression model	A small number of features

As illustrated by Table 2, the previous studies related to outcome prediction of ART methods are listed which have analyzed data using data mining and/or statistical methods. For this purpose, classifiers such as Decision Tree (DT), Logistic Regression (LR), Naïve Bayes (NB), K-Nearest Neighbors (K-NN), Support Vector Machines (SVM), Random Forest (RF), and Artificial Neural Networks (ANN) such as Multi-Layered Perceptron (MLP) and Radial Basis Function (RBF) have been used in the previous studies for predicting the clinical pregnancy after the complete cycles of different ART methods. A main drawback of the most of the considered previous studies is small volume of dataset and a few number of the considered features. Small dataset increases the risk of overfitting the trained models. Overfitting occurs when a model has good predictive ability for training dataset but shows poor performance for test dataset. Models with high overfitting property has lower generalization ability.

**Table 2:** List of the features engineered from the complex networks in this study

j = 1, 2, 3 where j = 1 indicates the index of the complex network (CN) made up of all training instances. J = 2 (or 3) are indices of complex networks consisting of all training instances excluding data records belonging to negative (or positive) class	
F_1_ = (node degree in CN_2_ – node degree in CN_3_) / node degree in CN_1_	(4)
F_2_ = (node weighted degree in CN_2_ – node weighted degree in CN_3_) / node weighted degree in CN_1_	(5)
F_3_ = (node closeness centrality in CN_2_ – node closeness centrality in CN_3_) / node closeness centrality in CN_1_	(6)
F_4_ = (node Eigen value centrality in CN_2_ – node Eigen value centrality in CN_3_) / node Eigen value centrality in CN_1_	(7)
F_5_ = (node betweenness centrality in CN_2_ – node betweenness centrality in CN_3_) / node betweenness centrality in CN_1_	(8)
F_6_ = (node clustering coefficient in CN_2_ – node clustering coefficient in CN_3_)/ node clustering coefficient in CN_1_	(9)
F_7_ = minimum length of the shortest path from the node in CN_2_ / minimum length of the shortest path from the node in CN_3_	(10)
F_8_ = the number of 2-hop neighbors of the node in CN_2_ / the number of 2-hop neighbors of the node in CN_3_	(11)
F_9_ = node degree in CN_2_ / node degree in CN_3_	(12)
F_10_ = node closeness centrality in CN_2_ / node closeness centrality in CN_3_	(13)
F_11_ = node Eigen value centrality in CN_2_ / node Eigen value centrality in CN_3_	(14)
F_12_ = node betweenness centrality in CN_2_ / node betweenness centrality in CN_3_	(15)
F_13_ = (normalized node degree in CN_2_—normalized node degree in CN_3_)/ max (normalized node degree in CN_2_, normalized node degree in CN_3_)	(16)
F_14_ = (normalized node closeness in CN_2_ – normalized node closeness in CN_3_)/ max (normalized node closeness in CN_2_, normalized node closeness in CN_3_)	(17)
F_15_ = (normalized node Eigen value in CN_2_ – normalized node Eigen value in CN_3_)/ max (normalized node Eigen value in CN_2_, normalized node Eigen value in CN_3_)	(18)
F_16_ = (normalized node betweenness in CN_2_ – normalized node betweenness in CN_3_)/ max (normalized node betweenness in CN_2_, normalized node betweenness in CN_3_)	(19)
F_17_ = (normalized node clustering coefficient in CN_2_ – normalized node clustering coefficient in CN_3_)/ max (normalized node clustering coefficient in CN_2_, normalized node clustering coefficient in CN_3_)	(20)

In this study, a dataset including the features of 11,255 IUI treatment cycles for 8360 couples is considered for IUI outcome prediction. Our dataset includes the couples' demographic characteristics, historical data about the patients' diseases, the clinical diagnosis, the treatment plans and the prescribed drugs during the cycles, semen quality, laboratory tests and the clinical pregnancy outcome. Considering the large number of couples and their corresponding IUI treatment cycles is a main advantage of this study compared to the considered previous studies.

On the other hand, most of the previous studies have considered the outcome prediction for IVF or ISCI. To the best of our knowledge, a few studies have been focused on predicting the outcome of IUI which have used clustering methods [9, 10] or regression analysis [11].

The previous studies which have been based on regression analysis only have considered the weights of the independent features to predict the overall pregnancy probability and they have not assessed the interconnection among the features [11–17]. Many previous studies have suffered from the lack of statistical power due to their small dataset [17, 18]. Also, the AUC performance of the previously proposed models for predicting IUI outcome have been low [12]. Therefore, it is required to improve the prediction performance by proposing novel methods and considering more data records.

Most of the considered previous studies have used single classifiers and/or RF as a simple ensemble classifier. Some previous studies have illustrated that the stacked models can improve the classification performance for other applications and other datasets [19–21]. Therefore, in this study, a novel stacked ensemble is designed and proposed for improving the performance of IUI outcome prediction.

The main aim of this study is to develop an automatic classification and feature scoring method to predict intrauterine insemination (IUI) outcome and ranking of the most significant features, based on the features describing the couples and their corresponding IUI treatment cycles. For this purpose, a novel approach combining complex network-based feature engineering and stacked ensemble (CNFE-SE) is proposed. Three complex networks are extracted considering the patients' data similarities. The feature engineering step is performed on the complex networks. The original feature set and/or the features engineered are fed to the proposed stacked ensemble to classify and predict IUI outcome for couples per IUI treatment cycle. Our study is a retrospective study of a 5-year couples' data undergoing IUI. Data is collected from Reproductive Biomedicine Research Center, Royan Institute describing 11,255 IUI treatment cycles for 8,360 couples.

The main novelty of this study lies in three folds including:

- Proposing a method for feature scoring and classification based on weighted complex networks and stacking ensemble classifiers
- Proposing feature engineering method based on complex networks
- Designing a novel stacked ensemble classifier for predicting IUI outcome

## Methods

**Fig. 1:**
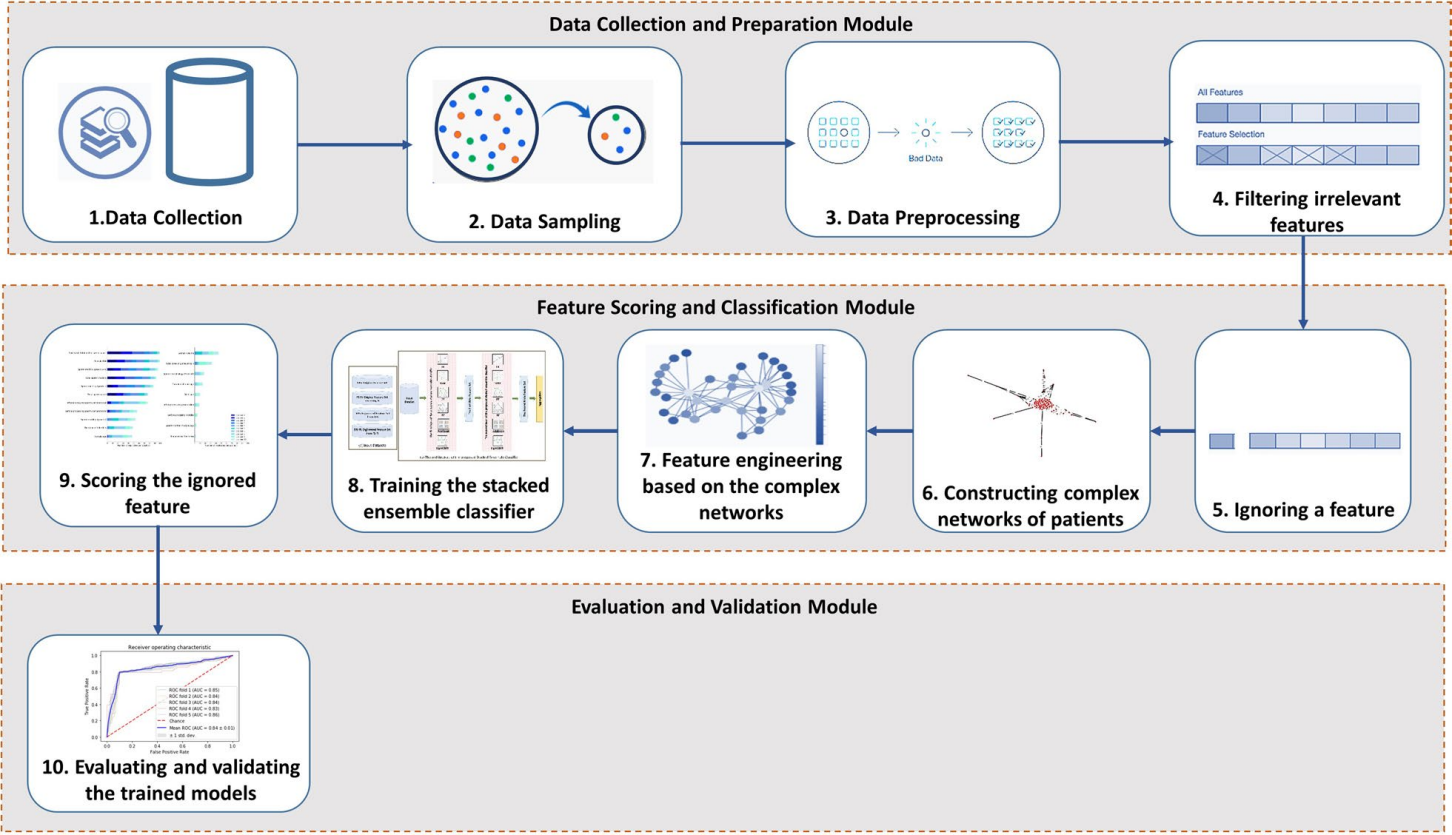
The main steps of the proposed method (CNFE-SE) for feature scoring and classifying the patients to predict IUI outcome

The main steps of the proposed approach combining complex network-based feature engineering and stacked ensemble (CNFE-SE) to predict the success of Intrauterine Insemination and ranking the features are illustrated in Fig. 1.

The main steps of the proposed method (CNFE-SE) as depicted in Fig. 1 include the modules for data collection and preparation, feature scoring and classification and finally model evaluation and validation. The first module consists of data collection, sampling from data, preprocessing the collected data and filtering irrelevant features. In the next module, ignoring a feature, constructing three complex networks from the patients, extracting features from the constructed complex networks, training the classifiers based on the extracted features and finally scoring the ignored feature are performed. The last module evaluates and validates the models trained in the previous module. More details about the mentioned tasks are described in the following subsections.

### Data collection

Our research is approved by the Institutional Review Board of the Royan Institute Research Center and the Royan Ethics Committee consistent with Helsinki Declaration with the approval ID of IR.ACECR.ROYAN.REC.1398.213. Anonymity and confidentiality of data were respected.

Dataset studied in this article is collected from Royan Institute, a public none-profitable organization, affiliated to the academic center for education, culture and research (ACECR) in Iran. It includes the features describing the patients having been treated by IUI method in the Infertility clinic at Royan Institute between January 2011 and September 2015.

In this retrospective study, a completed episode is defined as a sequence of treatment cycles resulting in positive clinical pregnancy or when the treatment with IUI is stopped. The inclusion criteria for the couples to be treated under IUI cycles were male factor, ovulatory disorders such as PCOS, hypothalamic amenorrhea, diminished ovarian reserve, combined causes, and unexplained subfertility. The couples' duration of infertility was at least 1 year. Male infertility was defined as the semen quality parameters lower than the standards determined by WHO including sperm concentration lower than 15 million/ejaculate, semen volume lower than 1.5 mL, and total motility lower than 40% [22]. The male partners with donor sperms, Varicocele, and semen samples with total motile sperm count lower than 1 × 10^6^ were excluded from being candidates for IUI treatment. Additionally, patients with anatomical and metabolic abnormalities, severe endometriosis and/or systemic diseases were excluded from our study.

11,255 IUI cycles related to 8,360 couples are considered in which the women age ranges from 16 to 47 with the average age of 29. This dataset contains 1,622 positive outcomes and 9,633 negative ones. Therefore, the overall pregnancy rate is 14.41% per completed cycle and 19.4% per couple. Each couple is treated for 1.31 ± 0.59 (mean ± Standard Deviation) IUI cycles which ranges from 1 to 7 cycle.

The features describe the couples' demographic characteristics, historical data about their diseases, the clinical diagnosis, the treatment plans and the prescribed drugs to the couples, male semen quality, laboratory tests and the clinical pregnancy outcome. The considered demographic features include age, body mass index (BMI), education level, consanguinity with spouse and some other features. The information about the history of the patients' subfertility consists of the duration and type of infertility, length of marriage and so on.

The types of feature values are numerical, binary, nominal and binominal types for 86, 152, 51 and 7 features, respectively. More details about the features is shown in Appendix 1.

**Fig. 2:**
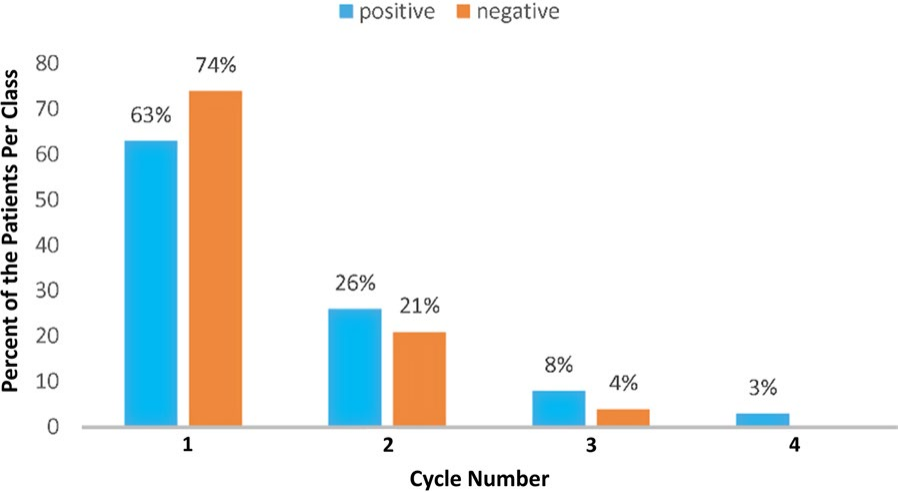
The ratio of positive and negative clinical pregnancy per treatment cycle

In the collected dataset, the majority of couples (almost 72%) have been treated for one cycle, 22% of couples have underwent two cycles, 5% of couples have been treated for three cycles, and less than 1% have been treated more than three cycles. The maximum number of cycles for treating a couple is seven. Figure 2 depicts the distributions of positive and negative clinical pregnancy rates for patients per treatment cycle.

As illustrated by Fig. 2, 63% of the couples belonging to the positive class (positive clinical pregnancy after completing the cycle) have been pregnant after the first treatment cycle. 26% of data records in the positive class have received positive outcome after the second cycle. Moreover, 74% of the couples in the negative class have been considered after the first cycle.

### Data sampling

Data should be randomly partitioned into training and test datasets with no overlapping among these two subsets. The models are trained on the training dataset and finally are evaluated by applying them to the test datasets.

K-fold cross validation (C.V.) is a common and popular sampling strategy used for this purpose. In this method, data is randomly divided into K disjoint equal-size subsets. Every time, one of these K subsets is considered as the test dataset and all (K-1) remaining subsets make the training one. The model is trained K times on K training datasets and applied to the corresponding test datasets to evaluate the performances of the trained models.

Before sampling from data, the features having missing value rate higher than 20% are removed from the study. Moreover, the patient records with high missing value rate (higher than 20%) are excluded from the study and then, fivefold C.V. is used for sampling from the collected dataset, in this study.

At first, dataset is partitioned into non-overlapping subsets D_1_, D_2_, …, D_K_ based on K-fold Cross Validation strategy. Then, the models are trained on K training datasets composed of all D_1_, …, D_K_ subsets excluding D_i_ for 1 ≤ i ≤ K. Therefore, the ith training dataset consists of all D_1_, …, D_K_ but D_i_ and the ith test dataset is D_i_. The ith training dataset is balanced using over-sampling strategy.

Moreover, a strategy for classification structural risk assessment is used named as A-Test which will be described in the evaluation and validation subsection with more details. The number of instances of positive and negative outcomes in each folder of fivefold is 324–325 and 1926–1927, respectively. therefore, the imbalance ratio of the training set in each of 5-folds is about 0.168.

### Data preprocessing

Preprocessing of data is one of the most essential steps in the knowledge discovery tasks. A previous study have stated that 80% of total time in data mining projects is allocated for data preparation and preprocessing step [23].

In the first step, the initial collected dataset includes almost 86,000 data records describing the partners and about 1,000 features. The data records describing one couple per IUI treatment cycle are aggregated to make our dataset. Thus, the aggregated dataset includes 11,255 data records and 296 features describing a couple during an IUI treatment cycle.

The nominal features are converted to dummy binary variables. If a nominal features has m different levels or values, it will be converted to (m-1) dummy binary variables. Therefore, instead of considering a nominal feature in the classification and feature ranking, its corresponding dummy binary variables are considered in the mentioned tasks.

The missing values for numeric and categorical features are imputed based on the average and the most frequent values, respectively [24]. All numerical and ordinal features are normalized using min–max normalization method and the nominal features are converted into dummy binary variables.

Outlier detection is performed in this study based on isolation forest method which has been proposed by Liu et al. [25] as an appropriate outlier detection method for high dimensional data. The hyperparameters of Isolation Forest including the number of estimators, maximum number of the samples, contamination coefficient, maximum number of the features, bootstrapping or not, and the number of jobs are tuned using grid search method. For evaluating the performance of Isolation Forest, its results are compared to other outlier detection methods such as One-class SVM with kernel of Radial Basis Function (RBF), boxplot analysis and expert's opinions. Three outliers are identified by this method and excluded from the study.

### Filtering irrelevant features

Since the aggregated dataset consists of many features, the irrelevant features can be removed to reduce the computational time required for processing and analyzing data. Thus, the features having very low correlation with the output feature or very high correlation with other input features are excluded from this study. The linear correlation coefficient between pairs of the features F_p_ and F_q_ are calculated as Eq. (1):

1 $$Corr\left( {F_{p} F_{q} } \right) = \mathop \sum \limits_{i} \frac{{\left( {F_{ip} - m_{p} } \right)\left( {F_{iq} - m_{q} } \right)}}{{\sqrt {\mathop \sum \nolimits_{j} \left( {F_{jp} - m_{p} } \right)^{2} } \sqrt {\mathop \sum \nolimits_{j} \left( {F_{jq} - m_{q} } \right)^{2} } }}$$

where F_x,p_ (F_x,q_) indicates the xth row of the feature F_p_ (F_q_) and m_p_ (m_q_) denotes the average of the feature F_p_ (F_q_), respectively.

If two features F_p_ and F_q_ have low (high) correlation, Corr (F_p_, F_q_) tends to zero (− 1 or + 1).

### Ignoring a feature

Breiman has proposed measuring the feature importance by mean decrease in accuracy (MDA) of random forest [26]. This study aims at ranking the features according to their predictive power for classifying the instances to positive or negative clinical pregnancy. For this purpose, all the steps 6–9 are performed by considering all the features excluding one feature each time and MDA for the trained proposed classifier is calculated on the validation dataset. MDA values show the amount of reducing the model accuracy after removing a feature. Therefore, the higher values of MDA indicate the higher predictive ability of the corresponding features.

### Constructing complex networks of patients

For modeling nonlinear data, complex networks are effective method [27]. Complex network is a weighted undirected graph G = (V, E, W), where V is the set of nodes, E denotes the set of edges e (v_i_, v_j_) between the pairs of the nodes v_i_ and v_j_ and W is the weights w (v_i_, v_j_) assigned to their corresponding edges e (v_i_, v_j_) of E.

Three complex networks are constructed from the training datasets and one data record which should be classified independent from it belongs to training or test dataset. The first one is comprised of all the training data records and one data record which should be classified as its nodes and is called CN1. The second and the third complex networks consist of one data record which should be classified and all training data records excluding the negative and positive classes and named as CN2 and CN3, respectively. If the considered data record belongs to training dataset, its class label is excluded from its corresponding complex networks.

In other words, the nodes of CN1, CN2 and CN3 are one data record which should be classified and all the training data records, positive labeled and negative labeled training data records, respectively. Therefore, for each data record, three complex networks are constructed.

An edge between node v_i_ and v_j_ is drawn if the distance between the input features of the ith and jth training data records is smaller than a user-defined threshold. For calculating the pairwise distance between data records, Euclidean distance function is used and can be calculated as Eq. (2):

2 $$Distance\left( {v_{i} v_{j} } \right) = \sqrt {\mathop \sum \limits_{p = 1}^{m} \left( {F_{ip} - F_{jp} } \right)^{2} }$$

where m is the number of the input features, F_i,p_ and F_j,p_ denote the pth input feature values for data records corresponding to v_i_ and v_j_.

The weight of the edge e(v_i_,v_j_) is calculated as Eq. (3):

3 $$w\left( {v_{i} v_{j} } \right) = \frac{{distance\left( {v_{i} v_{j} } \right)}}{{{\text{max}}\left( {distance\left( {v_{k} v_{h} } \right); v_{k} v_{h} \in V} \right)}}$$

### Feature engineering based on the complex networks

In this section, three complex networks per data record are constructed including the considered data record, all training instances as CN1 and all training instances excluding negative (positive) instances as CN2 (CN3). A simple intuitive hypothesis is that a node has more similarity with the training instances of its own class compared to the instances of the other class. Therefore, the node centrality in different complex networks CN1, CN2 and CN3 can be compared to classify the node. Features listed in Tables 3, 4 are defined based on this hypothesis.

**Table 3:** MDA values of top-20 features

Feature	Mean decrease in accuracy (MDA)
Post wash total motile sperm count	5.8
Female BMI	5.2
Sperm motility (grade a + b)	5
Total sperm motility	4.9
Sperm motility (grade c)	4.7
Total sperm count	4.5
After processing sperm concentration	4.3
Before processing sperm concentration	3.9
Sperm motility (grade d)	3.7
Male age	3.6
Semen volume	3.4
Duration of infertility	3.3
Total dose of gonadotropin	2.9
Female age	2.7
Duration of marriage	2.5
Sperm morphology (Amorph)	2.5
After processing progression	2.4
Before processing motility	2.3
Sperm normal morphology	2.2
Endometrial thickness	2.1

**Table 4:** Comparing the performance of CNFE-SE with other state of the art classifiers

Feature set	Classifier	Accuracy	Sensitivity	Specificity	AUC	*F *Score
All 296 features	RF	0.58 ± 0.01	0.69 ± 0.05	0.46 ± 0.06	0.58 ± 0.01	0.55 ± 0.05
	DT	0.55 ± 0.01	0.62 ± 0.04	0.49 ± 0.04	0.55 ± 0.01	0.55 ± 0.04
	NB	0.53 ± 0.01	0.79 ± 0.11	0.26 ± 0.12	0.54 ± 0.01	0.39 ± 0.11
	ANN	0.50 ± 0.01	0.54 ± 0.16	0.45 ± 0.16	0.50 ± 0.01	0.49 ± 0.16
	SVM	0.54 ± 0.01	0.28 ± 0.1	0.8 ± 0.09	0.56 ± 0.01	0.41 ± 0.05
	XGboost	0.55 ± 0.01	0.53 ± 0.03	0.56 ± 0.03	0.55 ± 0.01	0.54 ± 0.03
	LGBM	0.60 ± 0.01	0.59 ± 0.03	0.59 ± 0.01	0.64 ± 0.01	0.59 ± 0.02
	Adaboost	0.59 ± 0.01	0.69 ± 0.02	0.48 ± 0.02	0.60 ± 0.01	0.56 ± 0.02
	CNFE-SE without FE	0.71 ± 0.01	0.69 ± 0.01	0.73 ± 0.01	0.71 ± 0.01	0.71 ± 0.01
	CNFE-SE with FE	0.85 ± 0.01	0.79 ± 0.01	0.91 ± 0.01	0.84 ± 0.01	0.85 ± 0.01
Only most important features	RF	0.60 ± 0.02	0.69 ± 0.03	0.50 ± 0.02	0.59 ± 0.02	0.60 ± 0.02
	DT	0.57 ± 0.03	0.63 ± 0.01	0.54 ± 0.04	0.57 ± 0.02	0.58 ± 0.03
	NB	0.54 ± 0.01	0.52 ± 0.01	0.57 ± 0.01	0.54 ± 0.01	0.54 ± 0.01
	ANN	0.54 ± 0.01	0.55 ± 0.01	0.52 ± 0.01	0.53 ± 0.01	0.53 ± 0.01
	SVM	0.58 ± 0.01	0.51 ± 0.01	0.70 ± 0.01	0.60 ± 0.01	0.61 ± 0.01
	XGboost	0.58 ± 0.01	0.57 ± 0.01	0.59 ± 0.01	0.58 ± 0.02	0.58 ± 0.01
	LGBM	0.62 ± 0.02	0.61 ± 0.02	0.63 ± 0.03	0.62 ± 0.02	0.62 ± 0.02
	Adaboost	0.62 ± 0.01	0.69 ± 0.01	0.51 ± 0.01	0.61 ± 0.01	0.60 ± 0.01
	CNFE-SE without FE	0.72 ± 0.01	0.71 ± 0.01	0.74 ± 0.01	0.72 ± 0.01	0.72 ± 0.01
	CNFE-SE with FE	0.87 ± 0.01	0.82 ± 0.01	0.92 ± 0.01	0.87 ± 0.01	0.87 ± 0.01

Node degree is the number of its adjacent edges. Betweenness centrality for graph nodes have been introduced by Bavelas [28] and is calculated as Eq. (4). If a node lies in many shortest paths between pairs of nodes, its Betweenness centrality will be high. Nodes with high Betweenness centrality are the bridges for information flow.

4 $$Betweenness\left( {v_{i} } \right) = \mathop \sum \limits_{j < k} \frac{{number\,of\,the\,shortest\,paths\,between\,v_{j}\,and\,v_{k}\,passing\,v_{i} }}{{number\,of\,the\,shortest\,paths\,between\,v_{j}\,and\,v_{k} }}$$

Node closeness centrality measures the reciprocal of the sum of the length of the shortest paths between the node and all other nodes in the graph.

Node Eigen vector centrality is higher when the node is pointed to by many important nodes.

Clustering coefficient of a node is calculated as Eq. (5):

5 $$Clustering\,Coefficient\left( {v_{i} } \right) = \frac{{{\text{number\,of\,triangles\,connected\,to}}\,v_{i} }}{{{\text{number\,of\,triples\,centered\,around}}\,v_{i} }}$$

Since, the number of the instances are very high, the complex networks are partitioned into smaller communities to reduce the computational complexity for calculating the engineered features.

**Fig. 3:**
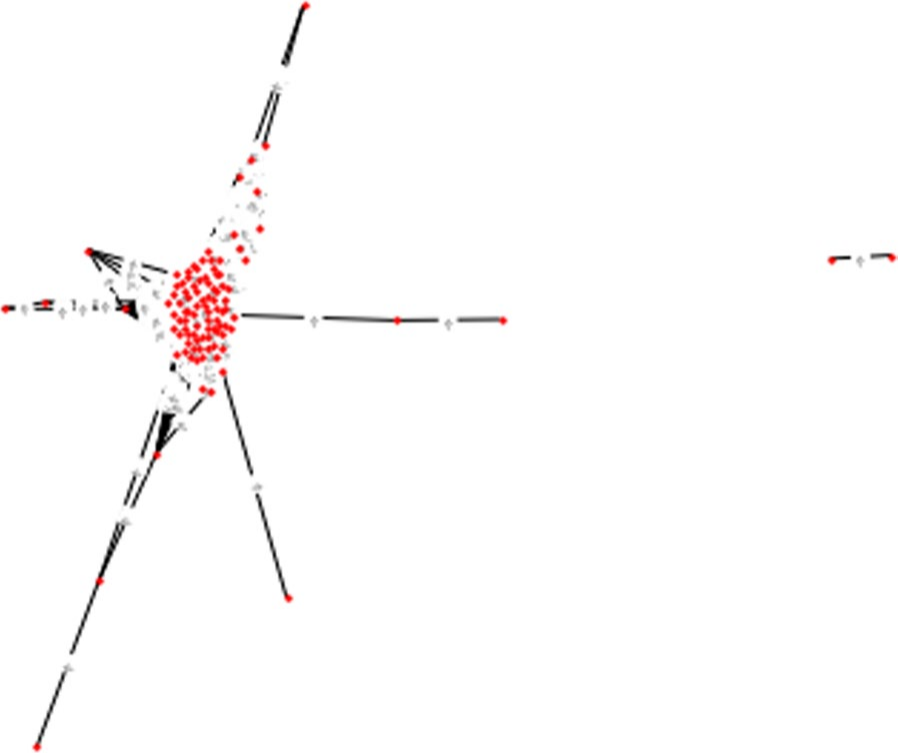
One complex network extracted from only 100 data records treated by IUI method

One complex network extracted from only 100 data records treated by IUI method as a sample is shown in Fig. 3.

**Fig. 4:**
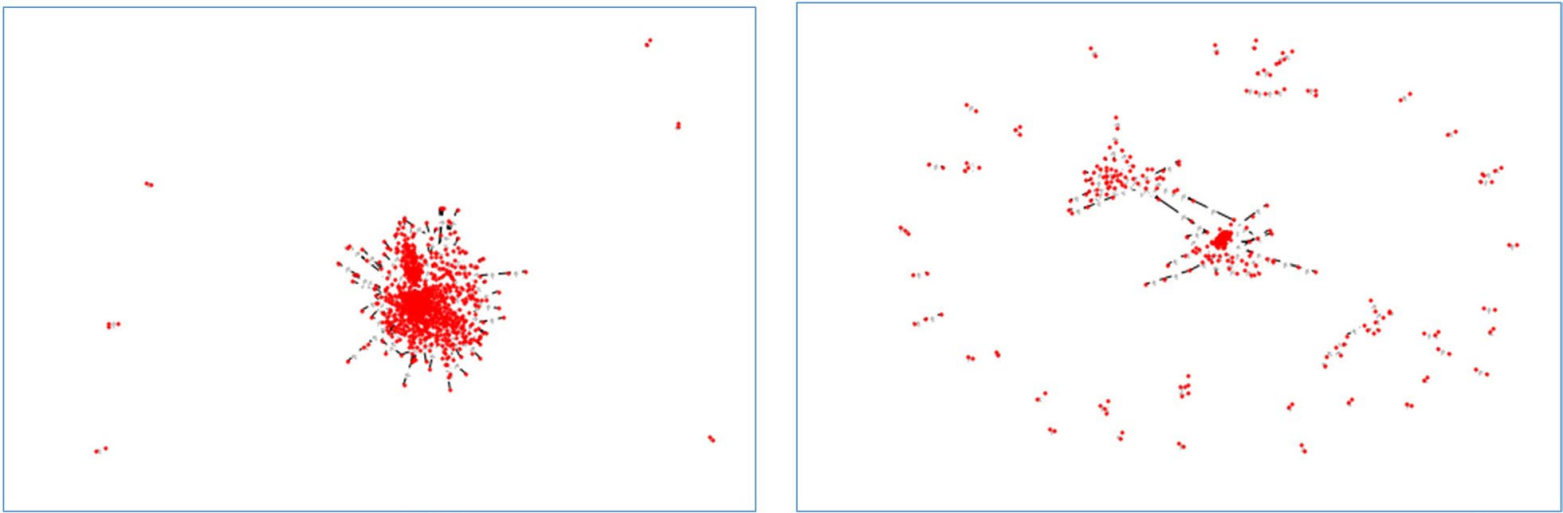
Two complex networks drawn from the positive training data samples by **a** threshold of 0.7 * average of the distance Matrix, **b** threshold of 0.5 * average of the distance matrix

Figure 4 depicts two complex networks of the same samples of positive instances drawn by different thresholds.

As shown by Fig. 4, reducing the threshold for keeping the edges in the complex network even with a small value lead to the network with more sparsity and more small-sized communities.

**Fig. 5:**
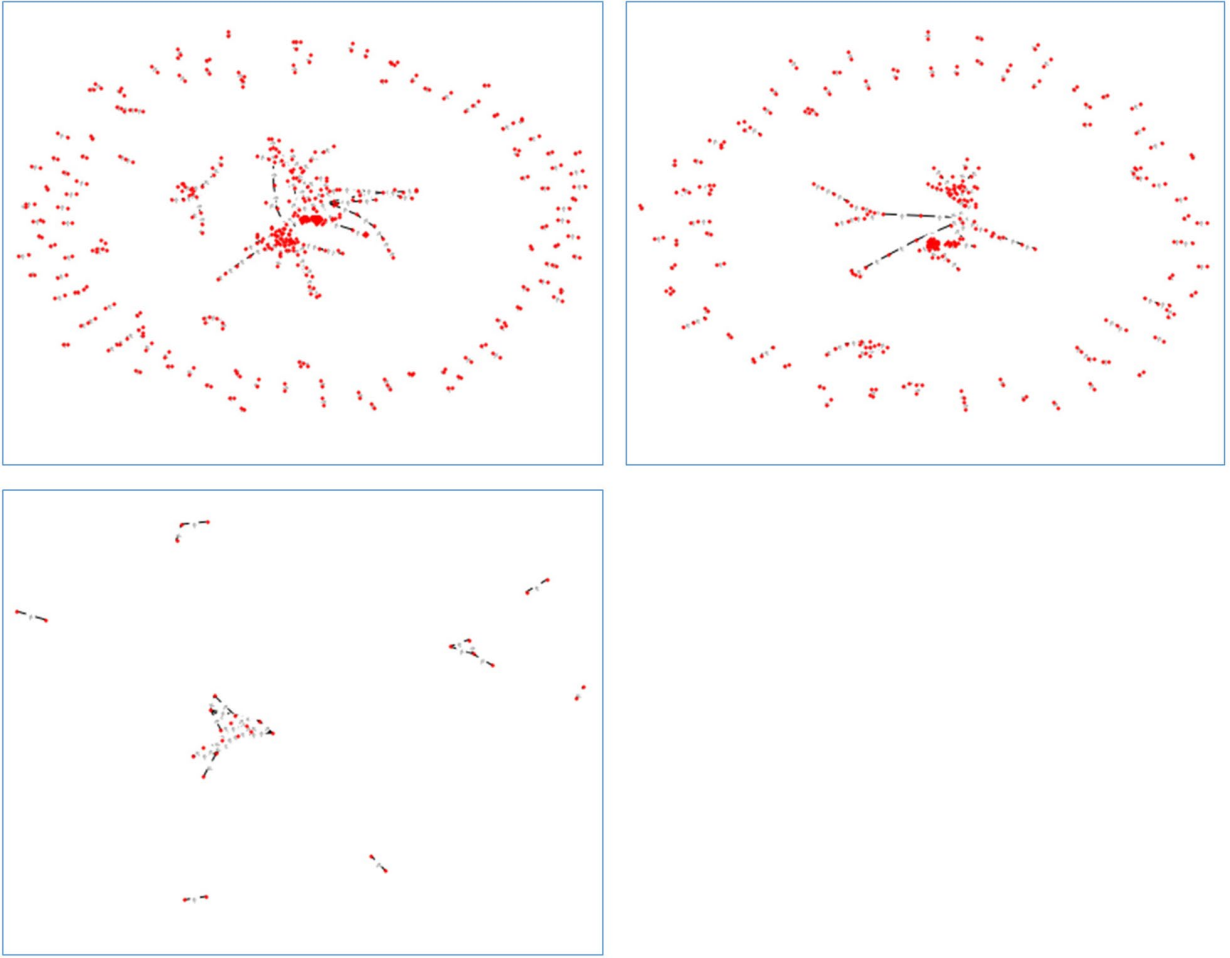
Three complex networks extracted from the samples of **a** both classes, **b** negative class, and **c** positive class with the same threshold

Figure 5 illustrates three complex networks from the samples of both classes, negative and/or positive classes.

As shown by Fig. 5, for the same thresholds, complex network considering the instances of both classes has the most density and the complex network from only positive instances has the most sparsity and consists of several small communities.

### Training the stacked ensemble classifier

Stacked ensemble classifier which is a scalable meta-modeling methodology has been first introduced by Wolpert in 1994 [29]. It has been inspired by neural networks whose classifiers have been considered as the nodes. Instead of a linear model, the stacked classifier can use any base classifier. The stacking operation has been performed by either a normal stacking or a re-stacking mode. In the normal stacking mode, the base classifiers in each layer use the output scores of the previous ones as the predictors similar to a typical feedforward neural network. The formula of normal stacking mode is written as Eq. (6):

6 $$f_{n} \left( {xV} \right) = V_{nk} \left( {f_{n - 1} \left( {xV_{n - 11} } \right) f_{n - 1} \left( {xV_{n - 12} } \right) , \ldots , f_{n - 1} \left( {xV_{{n - 1D_{n - 1} }} } \right)} \right)$$

where n indicates the nth layer of the stacked ensemble, x denotes a sample of a dataset, V presents a vector holding the neurons (the base classifiers), D is the number of hidden neurons through the nth hidden layer and finally, k is the kth neuron in the nth layer.

**Fig. 6:**
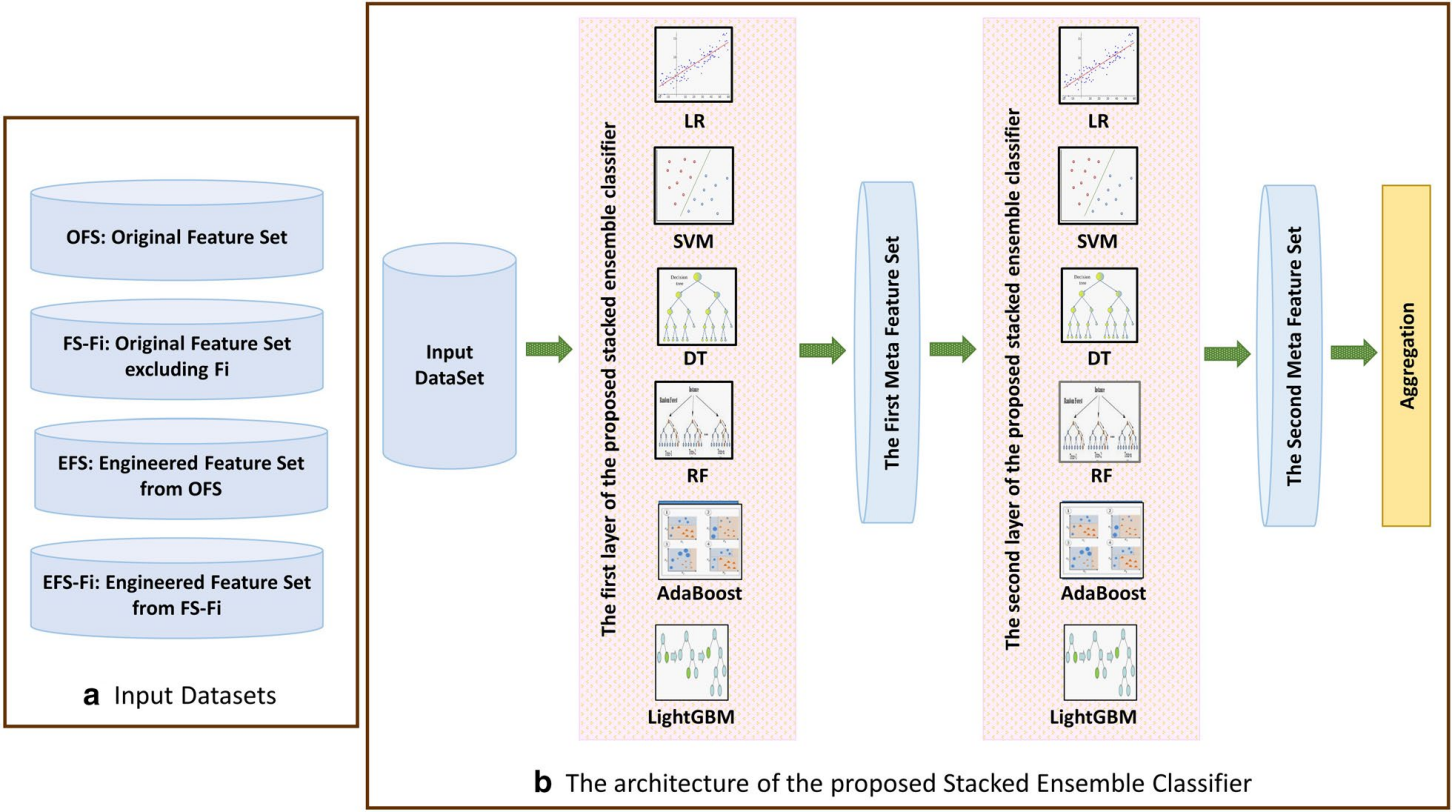
**a** Input datasets, and **b** the architecture of the proposed Stacked Ensemble classifier

Some previous studies have illustrated that the stacked models can improve the performance of the classification [20, 21, 30]. Therefore, in this study, a new stacked ensemble classifier is proposed and designed based on the normal stacking mode. In the beginning, some of the basic classifiers are trained, and those outperforming the others are selected to be considered as the base classifiers in the stacked ensemble layers. The architecture of the proposed stacked ensemble classifier is shown in Fig. 6.

As illustrated in Fig. 6, input dataset consists of the features in OFS, FS-Fi, EFS and/or EFS-Fi. Input dataset is fed to the base classifiers in the first layer of the proposed stacked ensemble classifier.

Several different classifiers are trained and verified. The classifiers for using in the ensemble layers of our proposed stacked ensemble classifier are chosen among different trained classifiers with different values of hyperparameters based on their accuracy and diversity on the validation dataset. A previous study has proposed a method to choose classifiers for ensemble learning based on accuracy and diversity which is used in this study for the same purpose. The pairwise diversity of the classifiers are calculated using Q statistic.

Logistic regression (LR) [31], support vector machines (SVM) [32], decision tree (DT) [33], random forest (RF) [26], Adaboost [34] and LightGradient Boosting Machine (LightGBM) [35] are the base classifiers chosen based on their accuracy and diversity in both ensemble layers.

LR, SVM with linear kernel and DT are appropriate classifiers for classifying linearly separable data. SVM with non-linear kernels, RF, Adaboost and LightGBM are ensemble classifiers which can classify nonlinearly separable data with high performance. All the mentioned classifiers can be trained fast. Therefore, they are chosen as the base classifiers of the proposed stacked ensemble classifier.

The hyperparameters of the classifiers are tuned based on grid search method and the best values for hyperparameters leading to the highest accuracy for validation dataset are considered for each classifier.

After training the base classifiers in the first layer, their outputs are considered as Meta features according to the normal stacking mode. The Meta features are fed into the base classifiers of the second layer for training them. Finally, the outputs of the base classifiers in the second layer are aggregated by weighted voting aggregation rule.

The weight of each base classifier is obtained by measuring its accuracy for classifying the validation dataset. The validation dataset is about 20% of the original training dataset which is excluded during the base classifiers' training in both layers.

Mathematical calculation is performed in this study to show the performance improvement obtained by stacked ensemble compared to traditional one-layer ensemble and the individual classifiers.

Without loss of generality, it is assumed that each base classifier in the first layer of stacked ensemble has the error rate of ε < 0.50. If the aggregation of the base classifiers is performed with bagging strategy which is the simplest aggregation method and uses majority voting, the error rate of the first ensemble layer (ε_L1_) can be calculated as Eq. (7):

7 $$\varepsilon_{L1} = \mathop \sum \limits_{{i = \left( {\frac{M}{2} + 1} \right)}}^{M} \left( {\begin{array}{*{20}c} M \\ i \\ \end{array} } \right)^{i} \varepsilon^{i} \left( {1 - \varepsilon } \right)^{M - i}$$

where M is the number of the base independent classifiers in the first ensemble layer. For misclassifying a data record using bagging strategy as the aggregation method, more than half of the base classifiers should misclassify the record. If it is assumed that i is the number of the base classifiers which misclassify the data record, i should be more than M/2 for misclassifying it with the first ensemble layer. For example, if M is 25, at least 13 base classifiers should misclassify data for erroneous classifying data in ensemble of these base classifiers. Now, if ε is 0.35 for each of 25 base classifiers, ε_L1_ will be 0.04. It shows the first layer of ensemble or traditional ensemble can improve the error rate of the single independent classifiers significantly.

Now, it is assumed that we have one more ensemble layer such as a two-layer stacked ensemble. Bagging strategy uses simple majority voting for classifying data as Eq. (8):

8 $$classLabel_{ensemble} \left( {r_{j} } \right) = \left\{ {\begin{array}{*{20}l} {Positive} \hfill & {if \mathop \sum \limits_{i = 1}^{M} \delta \left( {classLabel_{i} \left( {r_{j} } \right) = = Positive} \right) > \frac{M}{2}} \hfill \\ {Negative} \hfill & {otherwise} \hfill \\ \end{array} } \right\}$$

where r_j_ indicates the jth data record and i denotes the ith base classifier. As shown in Eq. (8), a simple decision tree or SVM with linear kernel can provide rules or find hyperplanes to classify data according to Eq. (8). Therefore, it can be shown that the performance of each base classifier in the second layer will not be worse than the simple bagging aggregation strategy used in the first ensemble layer.

This conclusion is true because each base classifier will try to find the hyperplane or rules to discriminate the training samples of two classes. But, bagging strategy uses simple majority voting. Furthermore, the input features (the first meta feature set as shown by Fig. 6) for the base classifiers of the second ensemble layer are the same as the input features fed to the bagging strategy in the first ensemble layer. These input features are the output class labels generated by the base classifiers in the first layer. Therefore, the error rate of each base classifier in the second ensemble layer would be at most ε_L1_.

The aggregation rule in the first ensemble layer is majority voting in the bagging strategy. The base classifiers try to separate the instances of different classes using linear or non-linear hyperplanes or rules. The input dataset for majority voting in the first ensemble layer is the first meta feature set. Therefore, the input of the majority voting rule and the base classifiers of the second ensemble layer is the same. The majority voting rule can be stated as Eq. (9) for the first meta feature set with M columns:

9 $$label_{MV} \left( {r_{j} } \right) = \left\{ {\begin{array}{*{20}c} {Positive} & {if \mathop \sum \limits_{i = 1}^{M} classLabel_{i} \left( {r_{j} } \right) > 0} \\ {Negative} & {otherwise} \\ \end{array} } \right\}$$

where MV is the majority voting strategy. Majority voting strategy is similar to using a hyperplane considering the equal coefficients for all of its input features as the separator of two classes.

The base classifiers try to find a best hyperplane for discriminating the instances of two classes. Therefore, their fitted hyperplane will not be worse than the hyperplane used with majority voting strategy. Thus, their performance will be more than or equal to the performance of the majority voting in the first ensemble layer. According to the Eq. (7), it is shown that the performance of the majority voting will be much better than the performance of the single classifiers in the first ensemble layer. Therefore, the performance of the single classifiers in the second ensemble layer will be better than the performance of the single classifiers in the first ensemble layer.

According to Eq. (7), if the bagging strategy is used for the second ensemble layer, the error rate of the second ensemble layer in the stacked ensemble would be ε_L2_ which can be calculated as Eq. (9):

10 $$\varepsilon_{L2} = \mathop \sum \limits_{{j = \left( {\frac{M2}{2} + 1} \right)}}^{M2} \left( {\begin{array}{*{20}c} {M2} \\ j \\ \end{array} } \right)\varepsilon_{b2}^{j} \left( {1 - \varepsilon_{b2} } \right)^{M2 - j} \le \mathop \sum \limits_{{j = \left( {\frac{M2}{2} + 1} \right)}}^{M2} \left( {\begin{array}{*{20}c} {M2} \\ j \\ \end{array} } \right)\varepsilon_{L1}^{j} \left( {1 - \varepsilon_{L1} } \right)^{M2 - j}$$

where M_2_ is the number of the base classifier in the second ensemble layer of the stacked ensemble and ε_b2_ is the error rates of the base classifiers in the second ensemble layer. As mentioned in the previous paragraph, the error rate of each base classifier in the second layer would be at most ε_L1_. Therefore, ε_b2_ will be not more than ε_L1_.

According to Eq. (7) and Eq. (9), the relationship among ε, ε_L1_ and ε_L2_ can be shown in Eq. (10):

11 $$\varepsilon_{L2} \ll \varepsilon_{L1} \ll \varepsilon$$

A previous study have demonstrated that adding more layers to stack ensemble can improve the classification performance in terms of accuracy and AUC [1].

Based on the obtained results, it can be shown that adding more layers to stacked ensemble can improve its performance. Although, adding more layers has higher burden of time complexity and memory usage, too.

There are a few studies considering the effect of the ensemble size or cardinality (the number of the base classifiers in the ensemble classifier) on the performance of the ensemble method [1, 2]. The previous studies have shown the ensemble size depends on the diversity of the base classifiers included in the ensemble and its aggregation rule [1, 2]. In addition, a previous study has examined different ensemble sizes including 10, 20, 50 and 100 classifiers for bioinformatics applications [3]. They have shown that the best ensemble size has been 50 but the ensemble size of 10 is sufficient to achieve to highly reasonable performance [3].

### Scoring the ignored feature

As mentioned in Sect. 1.5, MDA score is calculated for each feature and is considered as the feature importance score.

### Evaluating and validating the trained models

To evaluate the performances of the trained models, the performance measures for classification problems are used in this study including Accuracy, Sensitivity, Specificity and F-Score as shown in Eq. (11) -(14):

12 $$Accuracy = \frac{TP + TN}{N}$$

13 $${\text{Sensitivity}} = \frac{TP}{{TP + FN}}$$

14 $$Specificity = \frac{TN}{{TN + FP}}$$

15 $$F - Score = 2 \times \frac{Sensitivity \times Specificity}{{Sensitivity + Specificity}}$$

where TP and FP (TN and FN) indicate the number of instances in the positive (negative) classes which are classified correctly and incorrectly, respectively.

Moreover, the area under the curve (AUC) of the receiver operating curve (ROC) is considered.

In order to validate the results, the experiments are repeated 50 times, and each time the data is selected based on fivefold C.V.

A novel method named as A-Test has been proposed in a previous study to calculate the structural risk of a classifier model as its instability with the new test data [36]. A-test calculates the misclassification error percentage Γ_ζ,K_ for different K values using the balanced K-fold validation. In this study, the values of Γ_ζ,K_ will be reported for different classifiers and different feature sets. Γ_ζ,K_ is calculated as Eq. (15):

$$\Gamma_{{\zeta {\text{,K}}}} = \frac{100}{N}\left( {\begin{array}{*{20}c} {\mathop \sum \limits_{i = 1}^{N} \delta ((predictedLabel = Negative).\left( {realLabel = Positive} \right)) } \\ { + \mathop \sum \limits_{i = 1}^{N} \delta ((predictedLabel = Positive).\left( {realLabel = = Negative} \right))} \\ \end{array} } \right)$$

16 $$K = 2. \ldots .K_{max}$$

where K_max_ cannot be more than the size of the minority class. For estimating the structural risk of a classifier method, the average of the values of Γ_ζ,K_ is considered as Eq. (16):

17 $$ \Gamma _{\zeta }^{ \wedge }   = \frac{{\sum\nolimits_{{K = 2}}^{{K_{{\max }} }} {\Gamma _{{\zeta \cdot K}} } }}{{K_{{\max }} - 1}}$$

where Γ_ζ_^ ranges from 0 to 100% which higher values show higher risk of classification and lower values show the higher capacity and generalization ability of the model. Therefore, the lower values of Γ_ζ_^ are more desired.

## Experimental results

In this section, the features are ranked based on MDA obtained by ignoring them during the training of CNFE-SE. Then the partial dependencies between high-ranked features are discussed. Finally, the performance of the proposed model (CNFE-SE) is compared with other state-of-the-art classifiers.

### Ranking the significance of features

**Fig. 7:**
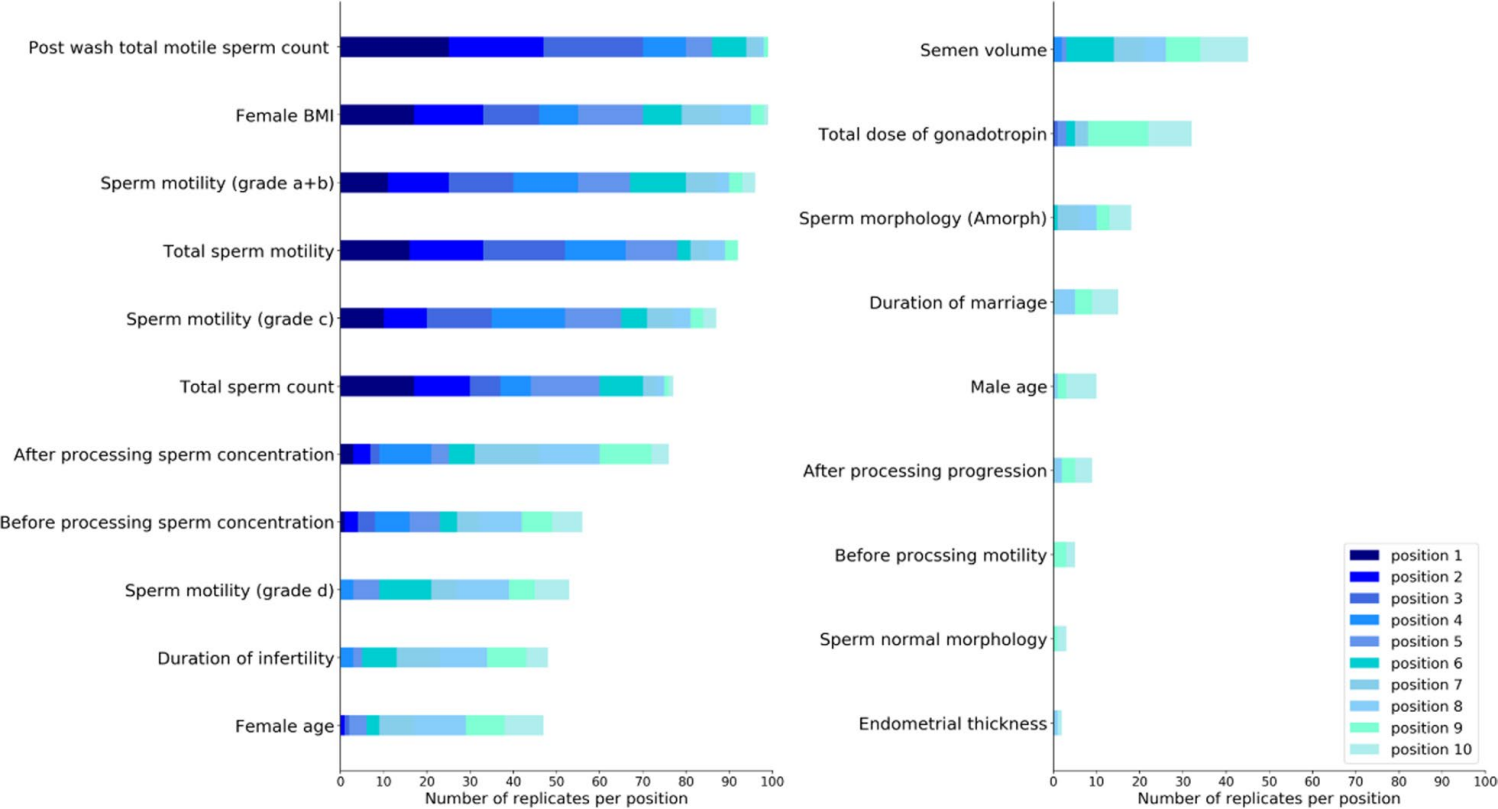
Overview of top features ranked based on CNFE-SE

Figure 7 represents top-20 important features with highest MDA score for IUI outcome prediction based on 50 repetitions of CNFE-SE training on different training samples. Post wash total motile sperm counts, female BMI, sperm motility grades a + b, total sperm motility and sperm motility grade c are high-ranked predictors of IUI outcome. Additionally, post-wash total motile sperm counts, female BMI, and total sperm counts are the features illustrated with dark blue colors in Fig. 7, have the highest repetitions as the first informative features. Generally, the variables related to the men's semen analysis parameters are high-ranked features in this study.

**Fig. 8:**
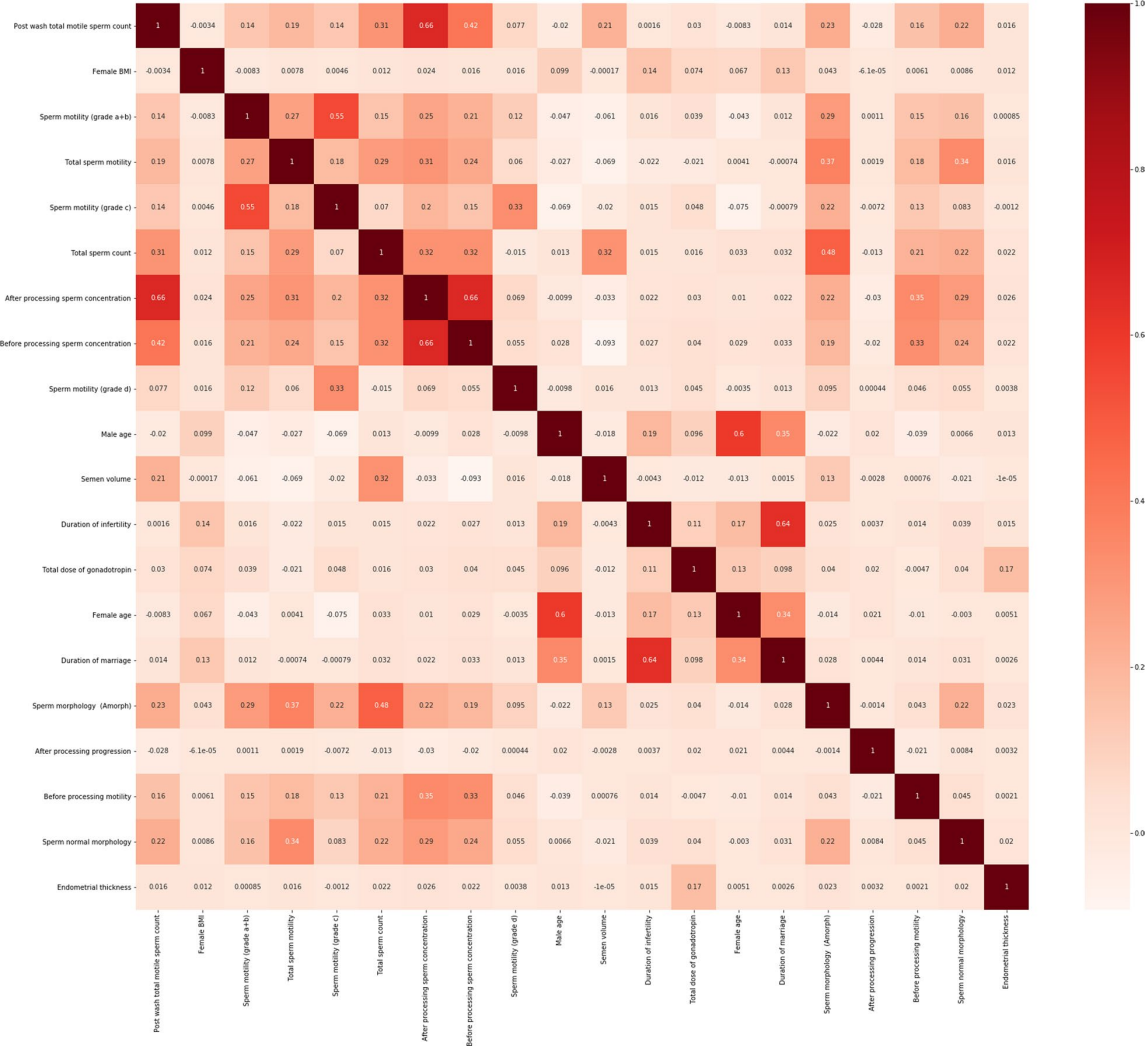
The pairwise correlation analysis of 20 most important features

The Pearson correlation coefficients are calculated among the top-20 important features, and Fig. 8 depicts the heat map of the correlation coefficients.

As shown by Fig. 8, the male semen parameters are positively correlated to each other, the more sperm concentration, the more total sperm count, and the more total motile sperm count. Also, couples' duration of infertility and duration of marriage are positively correlated.

**Fig. 9:**
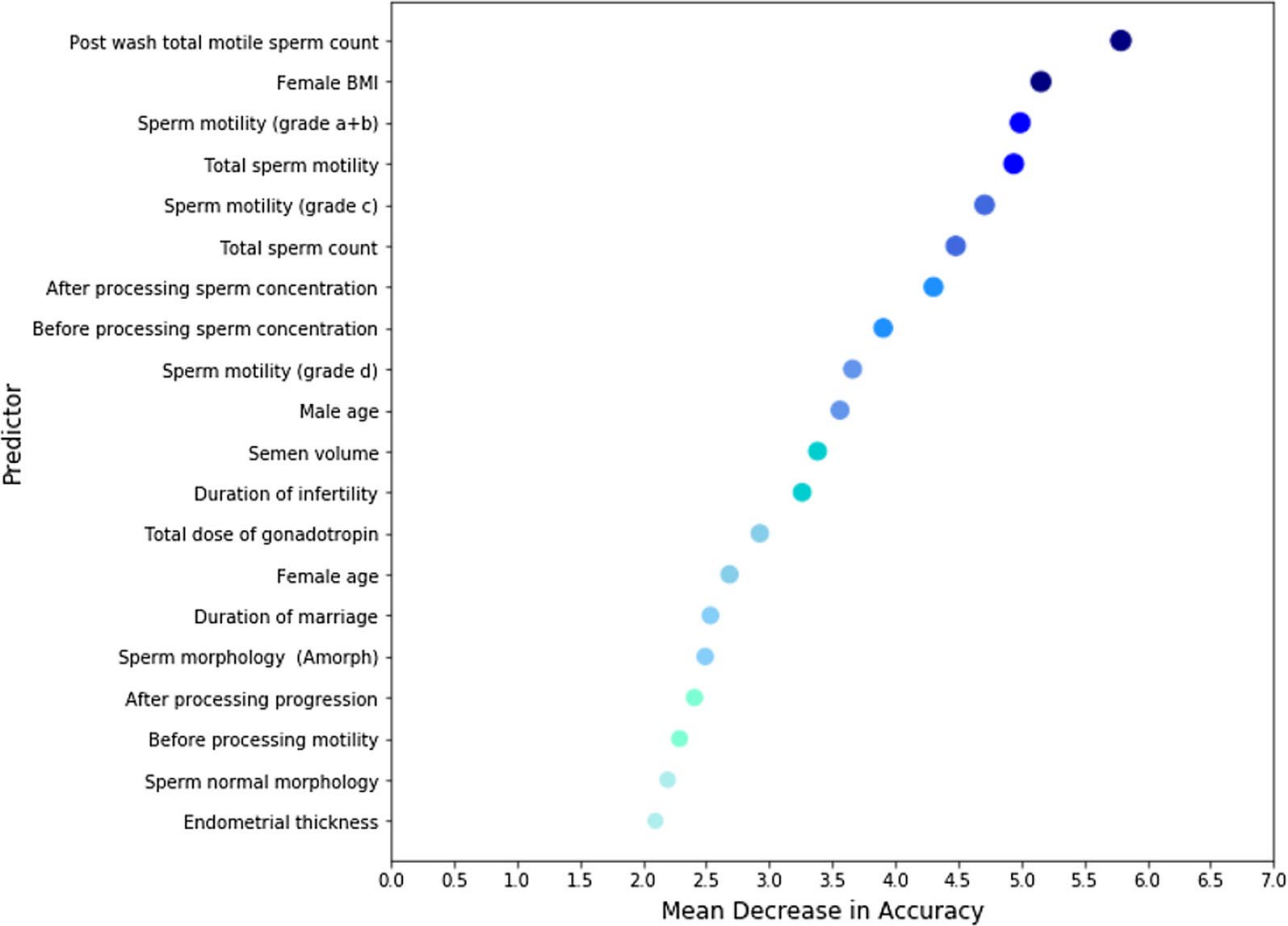
MDA values of top-20 features in this study

Figure 9 shows the exact values of MDA score for top-20 features in this study.

In addition, Table 3 lists MDA values of top-20 features.

### Partial dependency between the features

**Fig. 10:**
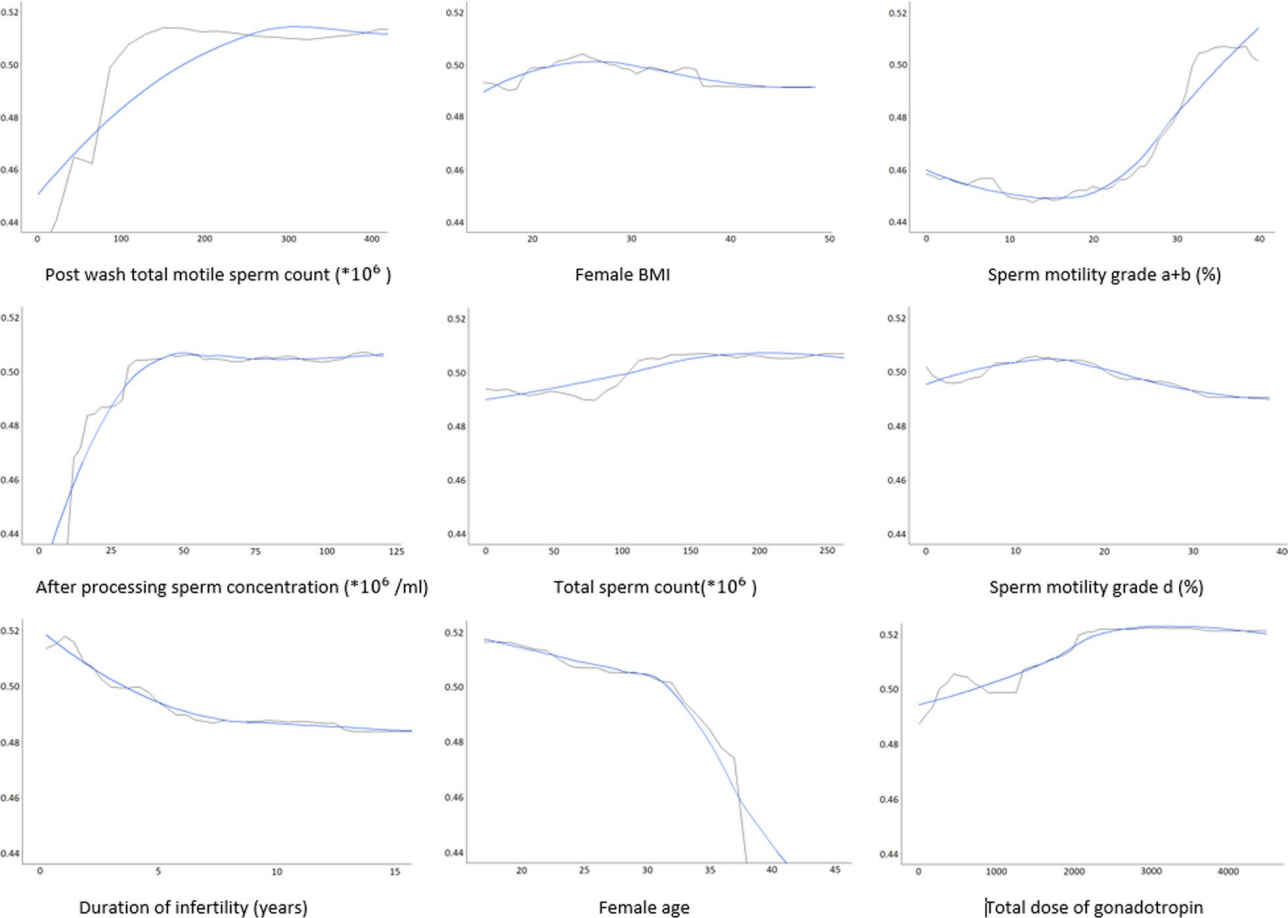
Partial dependency plots of nine features among the important features which the blue curves indicate locally weighted smoothing. It shows pregnancy variation obtaining from CNFE-SE (y-axis) as a function of a feature (x-axis) in IUI

Figure 10 depicts the partial dependency plots for the most important features. Partial dependency plots show whether a feature has a positive or negative effect on the response variable when the other ones are controlled. However, in order to interpret the graphs, we should note that changes in the clinical pregnancy probabilities in terms of the value of the features, even the most significant ones, are roughly small (the y-axis range is 0.44–0.52). Therefore, it is noteworthy that none of the features could individually and significantly alter the pregnancy rates more than 0.52. This finding underlines the value of the machine learning approach by determining the complicated association between individual predictors to make an effective classification model.

According to the results of the partial dependency plots as shown by Fig. 8, the clinical pregnancy rate has raised with increased number of post-wash total motile sperm counts and after processing sperm concentration. Also, when their values respectively vary upper than 100 million and 30 million spermatozoa per ml, the rate of pregnancy reaches its highest rate. In addition, the likelihood of IUI success increases through growing the number of total sperm counts which is mentioned in the previous studies, too [37].

### Comparing the performance of CNFE-SE with other state-of-the-art classifiers

Table 5 lists the performance measures for comparing CNFE-SE with other state of the art classifiers.

**Table 5:** The confusion matrix of CNFE-SE for total dataset

	Real positive	Real negative
Predicted positive	1296	860
Predicted negative	321	8772

Two different feature sets are considered as the input variables fed to the classifiers including all 296 features and only the most important features (top-20 features shown in Fig. 6). Moreover, CNFE-SE is trained and evaluated twice (one time without doing feature engineering (FE) and another time with performing feature engineering).

The models are executed and trained on different random training samples up to 50 times and the mean ± standard deviation values are depicted in Table 5. The CNFE-SE outperforms the compared models by AUC of 0.84 ± 0.01, sensitivity of 0.79 ± 0.01, specificity of 0.91 ± 0.01, and accuracy of 0.85 ± 0.01 when trains on all 296 features. Moreover, CNFE-SE has the superior performance when only 20-top features are fed to it as input variables with AUC of 0.87 ± 0.01, sensitivity of 0.82 ± 0.01, specificity of 0.92 ± 0.01 and accuracy of 0.87 ± 0.01. Our obtained results show that feature engineering and considering only 20-top features improve the performance of CNFE-SE.

Table 6 shows the confusion matrix of CNFE-SE for total dataset.

**Table 6:** Results of the A-Test: The values of Γ_ζ_^ and the minimum value of Γ_ζ_

Feature set	Classifier	Γ_ζ_^	Minimum of Γ_ζ_
All 296 features	RF	26.1	12.8
	DT	24.6	14.1
	NB	24.9	12.6
	ANN	25.5	14.5
	SVM	24.7	20.7
	XGboost	24.3	15.4
	LGBM	24.6	14.2
	Adaboost	25.8	12.7
	CNFE-SE without FE	16.9	11.7
	CNFE-SE with FE	11.3	6.8
Only most important features	RF	24.7	13.5
	DT	24.3	14.9
	NB	23.6	16.1
	ANN	24.6	15.8
	SVM	23.7	16.4
	XGboost	23.1	15.9
	LGBM	22.3	13.7
	Adaboost	21.5	13.6
	CNFE-SE without FE	16.1	11.3
	CNFE-SE with FE	10.9	6.2

**Fig. 11:**
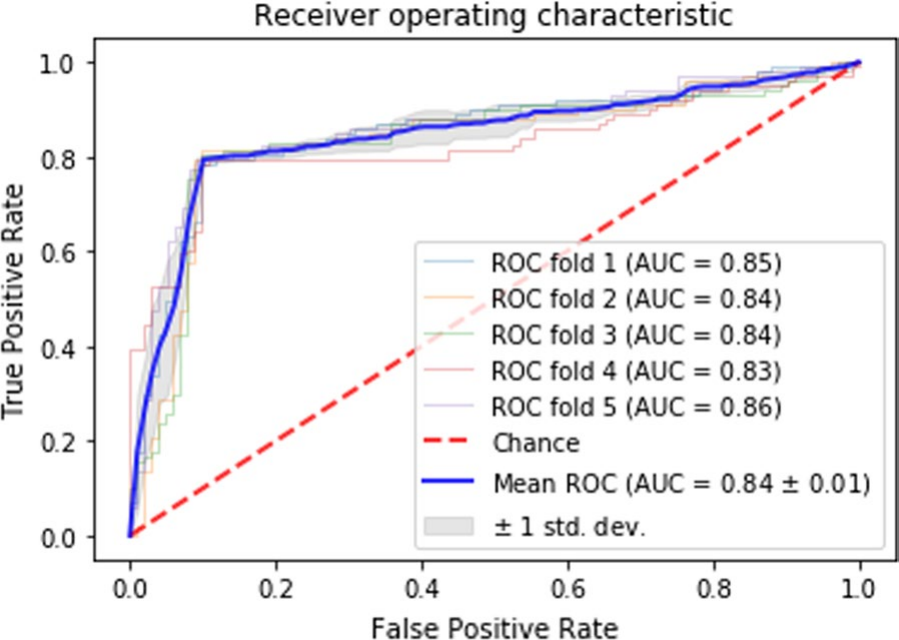
ROC curve for CNFE-SE trained with all features

Figure 11 depicts ROC curve for CNFE-SE trained with all features.

As shown by Fig. 11, AUC of CNFE-SE trained on all features is 0.84 ± 0.01. As illustrated by Table 5, the compared single classifiers show almost weak performances. The main reason is that the patients treated with IUI do not have complicated conditions and the leading cause of their infertility is idiopathic. Therefore, the data of the two classes have high similarity with each other, and their differentiation using single classifier is not an easy task. However, among these models, Light-GBM as one of state-of-the-art machine learning algorithms has the second best performance because it is a gradient boosting framework that uses tree-based learning algorithms and not only covers multi hyper-parameters but also has more focus on the accuracy of the results [35].

**Fig. 12:**
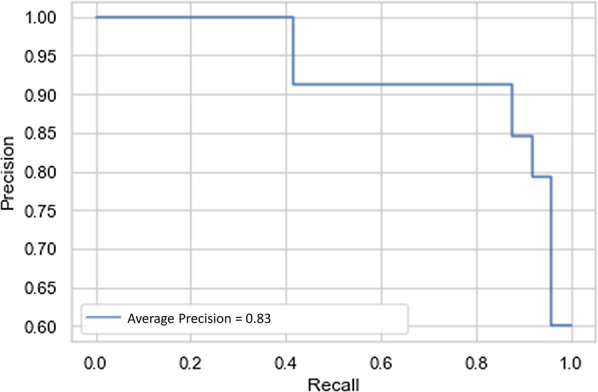
Precision-recall curves for CNFE-SE

When the classes are imbalanced, Precision-Recall curve is a useful instrument for the presentation of prediction success. A great area under this curve shows both high precision, which is related to low false-positive rate, and high recall, refers to low false-negative rate. Figure 12 indicates the precision-recall curves for CNFE-SE trained using top-20 features.

As shown in Fig. 12, CNFE-SE predicts both classes with highly reasonable performance.

Moreover, the results of A-test method for structural risk calculation for different combinations of feature sets and classifiers are shown in Table 7.

**Table 7:** The processing time details for our proposed method (FE: Feature engineering using complex network analysis)

Feature set	Model	Time for training (.sec)	Time for applying on one test data record (.sec)
All 296 features	CNFE-SE without FE	1751.62	21.47
All 296 features	CNFE-SE with FE	1902.88	28.13
top 20 features	CNFE-SE without FE	619.63	8.05
Top 20 features	CNFE-SE with FE	744.92	11.26

Lower values of Γ_ζ_^ and Γ_ζ_ shows lower risk of the classifier for classifying previously unseen records and the higher capacity and generalization ability of the model. Therefore, the feature set and classifier achieving the lower values of Γ_ζ_^ and Γ_ζ_ is more desired. As shown by Table 7, CNFE-SE trained using top-20 features has the superior performance based on A-Test results.

## Discussion

In the current study, among the various features that significantly affect the IUI outcome, the most potential predictors are female BMI and semen quality parameters. Semen data such as sperm count and motility are illustrated as the most prognostic factors in pregnancies, conceived by IUI and their association with IUI outcome have demonstrated in some previous studies [38]. Moreover, some previous studies have confirmed that semen descriptors, after the swim-up procedure have been more important than the ones before sperm washing process [39, 40]. Similarly, the percentage of motile sperm and its progression in the ejaculate have been known as significant predictors in IUI outcome prediction in the literature [41, 42]. Sperm motility grades a + b (progressive motility) and grade d (immotile sperms) are also determined in this study as potential predictive factors for a successful IUI [43]. Thus, if their corresponding values are more than 20% and less than 15%, respectively, the IUI success rate is higher.

Furthermore, the results of this study indicates that the IUI success rate is almost low when the female BMI is abnormal (BMI is lower than 20 or larger than 30). If female BMI is about 25 as the normal BMI value, the probability of pregnancy increases. This finding is mentioned in the previous studies, too [44].

Previous studies have shown that pregnancy rate could be reduced by increase in the female age [42, 45]. The present study identifies that the women older than 38 have a lower chance of successful IUI. However, Edrem et al. have not found the female age to be a prognostic factor in the prediction of IUI outcome [46].

As shown in Fig. 8, the duration of infertility inversely affects the fertility rate, and the decline in fecundity is acclaimed by some previous works, as well. Also, the previous studies have shown that when the couples' duration of infertility is less than six years, the pregnancy success rate is higher [47].

The total dose of gonadotropins is taken into account in this study as an important feature. Moreover, its significance has been considered recently, too [11]. This study identifies that the total dose of gonadotropin is positively correlated with the pregnancy rate. Moreover, other factors contributing to failure or success of IUI outcome according to this study's findings include semen volume, male age, sperm normal and amorphous morphology, duration of the marriage, and endometrial thickness which some of them have been demonstrated as the influential attributes in some previous studies [48–50].

Eventually, the CNFE-SE is trained using the 20 most important features and it yields surprisingly good performances (AUC = 0.87, 95% CI 0.86–0.88). It shows that the model carried out by these features, demonstrates a highly reasonable performance.

Some studies consider different patients' cycles as independent of each other, which may lead to a biased result. For example, they have considered the first cycle information [16, 51]. Our reanalysis of the primary cycle data revealed that the AUC performances of Light-GBM and CNFE-SE are 0.62 ± 0.01 and 0.84 ± 0.01, respectively, which does not change significantly when all the cycles are taken into account. Moreover, as shown in the materials and methods section, increasing the number of cycles augment the clinical pregnancy rate which are in line with the importance of this feature in subsequent IUI outcome [52, 53]. On the contrary, the variable cycle number has not identified as an important feature according to CNFE-SE feature scores. This finding may be due to the high number of data in the first cycle compared to the second, third and more cycles, which approximately 74% of the data belongs to the first cycle of IUI treatment.

Finally, our study has some restrictions. Some of the female hormonal tests including FSH, TSH, LH, and AMH have not been measured in all the patients before beginning IUI cycle, and therefore they are eliminated from the analysis due to their high missing value rate. At the Royan center, the patients who are entering the IUI treatment cycles are those who do not have complicated conditions, and the women's hormonal tests are usually normal. Moreover, the male BMI is excluded because of its high rate of missing values. The features describing the geographic information of couples’ habitats are removed from the study due to their low quality data entry.

**Fig. 13:**
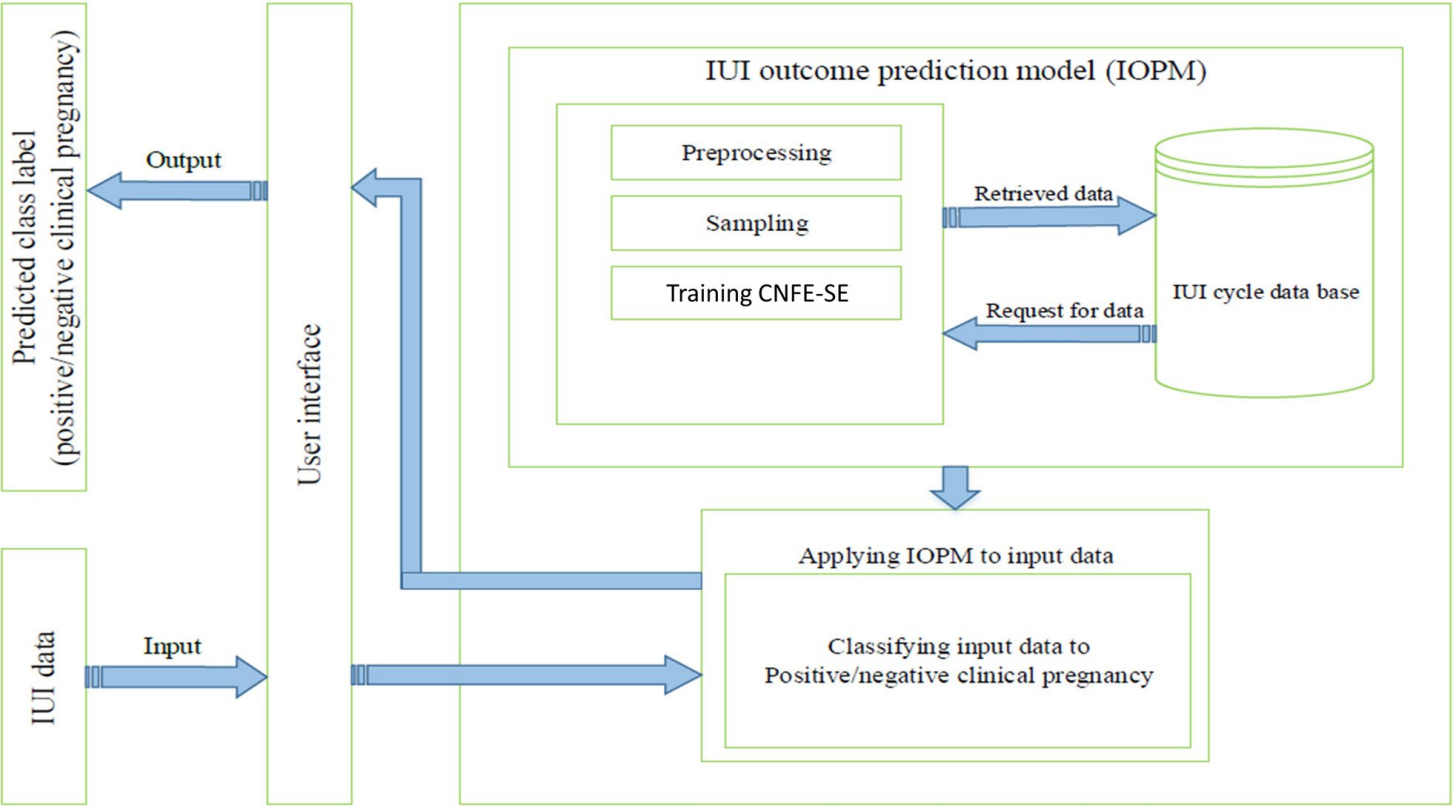
Schematic of our proposed medical decision support system for IUI outcome prediction

Currently machine learning algorithms has been increasingly employed in different medical fields [8]. Therefore, through using machine learning methods, we are able to predict the success or failure of the IUI cycle treatment outcome for each couple, based on their demographic characteristics and cycle information. In other words, our proposed CNFE-SE model shows superior performance among the compared state of the art classifiers. A decision support system (DSS) can be designed and implemented based on CNFE-SE. This DSS can help the physicians to choose other treatment plans for the couples and reduce patients' costs if their IUI cycle success rate is low. The schematic of this medical assistance system is shown in Fig. 13.

The proposed DSS is trained on the training dataset by CNFE-SE after preprocessing the collected dataset. After completing the training of CNFE-SE, every time a new data record is registered in the DSS, it can be classified by CNFE-SE into positive or negative outcome. The predicted outcome for the new data record can assist the physicians to decide to treat the couple with IUI method or not.

## Conclusion

In conclusion, the use of machine learning methods to predict the success or failure rate of the IUI could effectively improve the evaluation performances in comparison with other classical prediction models such as regression analysis. Furthermore, our proposed CNFE-SE model outperforms the compared methods with highly reasonable accuracy. CNFE-SE can be used as clinical decision-making assistance for the physicians to choose a beneficial treatment plan with regards to their patients’ therapy options, which would reduce the patients’ costs as well.

The experimental results in this study show that the most important features for predicting IUI outcome are semen parameters (sperm motility and concentration) as well as female BMI.

Some features which have been identified as good discriminative features for IUI outcome prediction in the previous studies are excluded from this study because of their high missing value rate. For example, some of the female hormonal tests including FSH, TSH, LH, and AMH are not routinely measured in all the patients before IUI and they are excluded from the study. It is proposed to augment dataset with data records without missing value in the mentioned features and consider the excluded features to CNFE-SE, and then try to rank the augmented feature set and evaluate the performance of the classifier.

On the other hand, some data records have noisy information which can reduce the performance of the classifiers. As future work, it is suggested that improving the robustness of CNFE-SE against the noisy data by including vote-boosting and other previously proposed methods for increasing the noise robustness of the classifiers. Moreover, the data is highly imbalanced which can have negative effect on the classifiers' performance. As another research opportunity, it is suggested that reducing the influence of data distribution per class by incorporating the advanced balanced sampling strategies.

Determining the optimal ensemble size is a challenging issue, yet. It is suggested that the impact of the ensemble size on the overall performance of stacked ensemble is studied in the future studies on different tasks and different datasets.

## Data Availability

Our study is a retrospective study of a 5-year couples' data undergoing IUI. Data is collected from Reproductive Biomedicine Research Center, Royan Institute for 8,360 couples who underwent 11,255 IUI cycles were included. But, we are not allowed to share the original dataset because of the privacy and security issues.

## Appendix

See Tables 8 and 9.

**Table 8:** Feature description for our dataset

Variable	Variable levels	Variable type
Endoscopy-hysteroscopy	0, 1st Hysteroscopy, 1st Hysteroscopy | 2nd Hysteroscopy, 1st Hysteroscopy | 2nd Hysteroscopy | 3rd Hysteroscopy,, 3rd Hysteroscopy | 1st Hysteroscopy, 4th Hysteroscopy | 3rd Hysteroscopy | 1st Hysteroscopy | 2nd Hysteroscopy	Nominal
Endoscopy-laparoscopy	0, 1st laparoscopy, 1st laparoscopy | 2nd laparoscopy, 2nd laparoscopy	Nominal
Endocrine disorder-pituitary	0, acromegaly, Prolactinoma	Nominal
History-Habit	0, Alcohol, Alcohol | smoking, smoking	Nominal
Medical HX-neuro psychological	0, Anxiety, depression, depression | Epilepsy, Epilepsy, Psychotic disorder	Nominal
Medical HX-GI	0, Appendicitis, IBD, IBS, IBS | Peptic ulcer, Peptic ulcer	Nominal
Medical HX-immunologic disorder	0, Arthritic rheumatoid, lupus	Nominal
Medical HX-respiratory disease	0, Asthma, Bronchiectasis, bronchitis, bronchitis | Asthma	Nominal
Common surgery-orchiopexy	0, Bilat, Bilat | Left | Right, Left, Right, Right | Left	Nominal
Common surgery-varicocelectomy	0, Bilat, Left, Left | Bilat | Right, Left | Right, Right	Nominal
Common surgery-hernia	0, Bilat, Left, Left | Right, Left | Right | Bilat, Right	Nominal
Common surgery-hydrocele	0, Bilat, Left, Right	Nominal
History-nipple discharge	0, Bilateral, Bloody, Unilateral	Nominal
Current drug-antihyperprolactinemia	0, Bromocriptin, Bromocriptin | Dostinex, Dostinex	Nominal
Medical HX-infection disorder	0, Brucellosis, Hepatitis A, Hepatitis B, Hepatitis C, Herpes, TB	Nominal
Endocrine disorder-thyroid	0, cancer, Hyper, Hyper | Hypo, Hypo, Nodule	Nominal
Medical HX cardiovascular disorder	0, DVT, Heart failure, Hypertension, Hypertension | MVP, MVP	Nominal
Menstrual criteria-with drug	0, Estrogen, OCP, Progesterone, Progesterone | Estrogen, Progesterone | OCP	Nominal
History-pelvic infection HX	0, Frequent discharge, History Of PID	Nominal
Current drug-antidiabetic	0, Glucophage, Metformin, Metformin | Glucophage, Metformin | Paraovarian cyst, Paraovarian cyst	Nominal
LT G	0, II, II | III, II | Time of Injection, III, Time of Injection	Nominal
RT G__1	0, II, II | Time of Injection, Time of Injection	Nominal
LT G__1	0, III, Time of Injection	Nominal
Medical HX-blood disorder	0, Iron anemia, Major thalassemia, minor thalassemia	Nominal
Common surgery-orchiectomy	0, Left, Right	Nominal
Current drug-thyroid drugs	0, Levothyroxin, Levothyroxin | metimazole, metimazole, PTO	Nominal
Past-irreg	0, Oligomenorrhea, Poly menorrhea	Nominal
Medical HX-renal disease	0,Recurrent infection, Renal anomaly, Renal stone	Nominal
Diabetes-1st degree relative	0,Type I, Type I | Type II, Type II	Nominal
Endocrine disorder-D.M	0, type1, type2	Nominal
diagnosis-Uterine F	adenomyosis, Anomaly, Asherman, Myoma, Negative, Unexplained (thin endometrium)	Nominal
diagnosis-ovulatory F	Age factor, Diminished ovarian reserve, Endocrine problem, Hypothalamic hypogonadism, Negative, PCOS	Nominal
Therapeutic-agglutination	all, few, most, none, some	Nominal
Specimen characteristics-color	Amber-Yellow, gray | yellow, Grey, Light-Yellow, Milky, White, White–Grey	Nominal
Therapeutic-type of sampling	Coitus, Coitus | Masturbation, Masturbation, Retrograde ejaculation, Retrograde ejaculation | Coitus	Nominal
Therapeutic sperm preparation -technique	Discontinuous gradient, Pure sperm | Discontinuous gradient, Swim up, Swim up | Discontinuous gradient	Nominal
diagnosis-recurrent abortion	Endocrine, Male, Negative, Thrombotic, unexplained	Nominal
Tuboperitoneal type	Endometriosis, EP Hx, hydrosalipinx, Idiopathic, Negative, Post surgery	Nominal
diagnosis-unexplained	Endometriosis, Idiopathic, Negative	Nominal
diagnosis-other diagnosis	Genetic, Impotency, Incomplete data, Negative, other, poor obstetric outcome, sex selection, Vaginismus	Nominal
Menstrual criteria-amount	hypermenorrhea, Hypo menorrhea, Normal	Nominal
IUI clinic-IUI catheter	INDOVASIVE, Other, INDOVASIVE | Other, Catheter Impex, ORI	Nominal
Past-type	more than 8 levels	Nominal
Surgical HX-non gynecologic surgery	more than 8 levels	Nominal
DRUG HX-past drug	more than 8 levels	Nominal
Male medical information-exposures	more than 8 levels	Nominal
Male medical information-drug Hx esp	more than 8 levels	Nominal
Coded after processing-progression	more than 8 levels	Nominal
Coded before processing-progression	more than 8 levels	Nominal
Therapeutic-viscosity	Normal, Somewhat Thick, Thick, Thin, Very thick	Nominal
Specimen characteristics-viscosity	Normal, Somewhat Thick, Thick, Very Thick	Nominal
Family history endocrine disorder-thyroid	NO, YES	Binominal
Male medical information-scrotum	Ab, NL	Binominal
Specimen characteristics-collect type	C, M	Binominal
Therapeutic-place of sampling	In, Out	Binominal
Therapeutic-SPLIT Ejaculation	NO, YES	Binominal
Menstrual Criteria-Interval	Normal (22–35 days), Abnormal (< 21, > 36 days)	Binominal
Infertility type	primary, secondary	Binominal
Relationship-First cousin	0,1	Binary
Relationship-distant relationship	0,1	Binary
No relationship	0,1	Binary
Diagnosis-Male factor	0,1	Binary
Past-Reg (menstruation)	0,1	Binary
Current-Reg (menstruation)	0,1	Binary
Irreg-Oligomenorrhea	0,1	Binary
Irreg-Poly menorrhea	0,1	Binary
Cycle-Amenorrhea	0,1	Binary
Amenorrhea-Primary	0,1	Binary
Amenorrhea-Secondary	0,1	Binary
Menstrual Criteria-IMB	0,1	Binary
Coital HX-Lubricant	0,1	Binary
Coital HX-PCB	0,1	Binary
Coital HX-Vaginismus	0,1	Binary
Coital HX-Impotency	0,1	Binary
History-Cytotoxic Therapy	0,1	binary
Allergy-food	0,1	Binary
Allergy-seasonal	0,1	Binary
Allergy-Skin	0,1	Binary
Allergy-respiratory	0,1	Binary
Family history-infertility	0,1	Binary
Family history-recurrent abortion	0,1	Binary
Family history-POF	0,1	Binary
Family history-hearing disorder	0,1	Binary
Family history-TB	0,1	Binary
Family history-mental retardation	0,1	Binary
Cardio vascular-myocardial infarction	0,1	Binary
Cardio vascular-HTN	0,1	Binary
Respiratory disorder-Asthma	0,1	Binary
Thalassemia-minor	0,1	Binary
Type of cancer-breast cancer	0,1	Binary
Type of cancer-colon cancer	0,1	Binary
Type of cancer-leukemia	0,1	Binary
Type of cancer-uterus cancer	0,1	Binary
Type of cancer-lung cancer	0,1	Binary
Family history-epilepsy	0,1	Binary
Antihyperprolactinemia-Bromocriptin	0,1	Binary
Antihyperprolactinemia-Dostinex	0,1	Binary
Current drug-Anti Depression	0,1	Binary
Current drug-Anti hypertension	0,1	Binary
Current drug-Anticoagulant	0,1	Binary
Current drug-Folic acid	0,1	Binary
Current drug-Estrogen	0,1	Binary
Current drug-Progestron	0,1	Binary
Current drug-Ferrous sulphate	0,1	Binary
Current drug-Multi vitamin	0,1	Binary
Physical Exam-hirsutism	0,1	Binary
Type of pregnancy-Clinical pregnancy (class variable)	0,1	Binary
Minimal stimulation-Gonadotropin	0,1	Binary
Minimal stimulation-Letrosol	0,1	Binary
Minimal stimulation-Clomiphen	0,1	Binary
Type-Cetrorelix	0,1	Binary
HCG.IU_5000	0,1	Binary
HCG.IU_10000	0,1	Binary
HCG.IU_15000	0,1	Binary
Buserelin_0.5 cc	0,1	Binary
Ovitrelle_250 mgr	0,1	Binary
Ovitrelle_500 mgr	0,1	Binary
Type-Fostimon	0,1	Binary
Type-Gonal.F	0,1	Binary
Type-Menopour	0,1	Binary
Type-HMG	0,1	Binary
Type-Merional	0,1	Binary
Type-Bravelle	0,1	Binary
Before Stimulation-OCP	0,1	Binary
Before Stimulation-Estradiol	0,1	Binary
Cotreatment-Aspirin	0,1	Binary
Adjuvant therapy-Acid folic	0,1	Binary
Right-PCO	0,1	Binary
Right-Cyst	0,1	Binary
Left-PCO	0,1	Binary
Left-Cyst	0,1	Binary
Endometrial Texture-Three line	0,1	Binary
have you married before-yes	0,1	Binary
did you have any child-no	0,1	Binary
Common surgery-T.BX	0,1	Binary
Common surgery-Vasectomy	0,1	Binary
Uncommon surgery-Urethral stricture	0,1	Binary
Uncommon surgery-Brain surgey	0,1	Binary
Medical history-Mumps	0,1	Binary
Medical history-Hypertension	0,1	Binary
Medical history-Veneral Dz	0,1	Binary
Medical history-Allergy	0,1	Binary
Medical history-D.M	0,1	Binary
Medical history-M orchitis	0,1	Binary
Medical history-Test Pain	0,1	Binary
Medical history-TB	0,1	Binary
Medical history-Epididimorchitis	0,1	Binary
Medical history-UDT	0,1	Binary
Medical history-UTI	0,1	Binary
Cigarette-occasionally	0,1	Binary
Cigarette-quarter pack per day	0,1	Binary
Cigarette-half pack per day	0,1	Binary
Cigarette-one pack per day	0,1	Binary
Cigarette-two packs per day	0,1	Binary
Cigarette-three packs per day	0,1	Binary
Alcohol-occasionally	0,1	Binary
Alcohol-one per week	0,1	Binary
Opium-occasionally	0,1	Binary
Opium-one per day	0,1	Binary
Marital status of brothers-Married	0,1	Binary
Marital status of brothers-New couples	0,1	Binary
Family HX of infertility-Do your brothers have any children	0,1	Binary
Right testicle Size_less than 1	0,1	Binary
Right testicle Size_1-1.5	0,1	Binary
Right testicle Size_1.5–2	0,1	Binary
Right testicle Size_2-2.5	0,1	Binary
Right testicle Size_2.5–3	0,1	Binary
Right testicle Size_3-3.5	0,1	Binary
Right testicle Size_3.5–4	0,1	Binary
Right testicle Size_more than 4	0,1	Binary
Left testicle size_less than 1	0,1	Binary
Left testicle size_1-1.5	0,1	Binary
Left testicle size_1.5–2	0,1	Binary
Left testicle size_2-2.5	0,1	Binary
Left testicle size_2.5–3	0,1	Binary
Left testicle _3-3.5	0,1	Binary
Left testicle size_3.5–4	0,1	Binary
Left testicle size_more than 4	0,1	Binary
Vas-RT	0,1	Binary
Vas-LT	0,1	Binary
RT-NL	0,1	Binary
RT-Ab	0,1	Binary
LT-NL	0,1	Binary
RT-G	0,1	Binary
plan-med treatment	0,1	Binary
Vitamin-Vitamin C	0,1	Binary
Vitamin-Vitamin E	0,1	Binary
Plan-S.A	0,1	Binary
Plan-Imaging	0,1	Binary
Plan-H.A	0,1	Binary
Plan-CW	0,1	Binary
Plan-KARYO	0,1	Binary
Plan-AZF	0,1	Binary
Plan-TESE	0,1	Binary
Plan-Wives visit by Gyn required	0,1	Binary
Plan-Varicocelectomy	0,1	Binary
Plan-sperm Freezing	0,1	Binary
Plan-PC.U.A	0,1	Binary
Plan-genetic consult	0,1	Binary
Plan-endocrine consult	0,1	Binary
Plan-DFI	0,1	Binary
Plan-stop smoking, alcohol, opium	0,1	Binary
Plan-Occupation hygiene	0,1	Binary
Plan-Low weight	0,1	Binary
Plan-Others	0,1	Binary
Morphology-Amorph	0,1	Binary
Morphology-giant head	0,1	Binary
Morphology-pin head	0,1	Binary
Morphology-round head	0,1	Binary
Morphology-cytoplasmic droplet	0,1	Binary

**Table 9:** Summary statistics for the features of our dataset

Feature name	Min	First Q	Median	Third Q	Max	Mean	SD
Time diff between cycles	0	0	0	2	6.1	1.59	4.82
	0	0	0	1	6.9	1.25	3.83
Age_x	17	25	28	31	43	28.29	4.29
	16	26	29	32	47	28.97	4.68
Family demographic information (Education_x)	1	3	3	5	7	3.43	1.17
	1	3	3	5	7	3.4	1.22
Duration of marriage (years)	0.42	4	6	7	23	6	2.94
	0.25	4	6	7.5	26	6.31	3.19
Duration of infertility (Years)	0.25	2.5	4	5	15	4.36	2.62
	0.17	2.84	4.5	6	19	4.66	2.85
Menstrual HX (menarche old year)	9	13	13	14	27	13.35	1.46
	9	13	13	14	27	13.29	1.42
Current contraception duration	0	0	0	0	3	0.02	0.17
	0	0	0	0	8	0.01	0.19
Summary-Gravida	0	0	0	0	6	0.29	0.66
	0	0	0	0	6	0.26	0.61
Summary-para	0	0	0	0	3	0.08	0.31
	0	0	0	0	4	0.08	0.3
Abortion-early	0	0	0	0	4	0.17	0.48
	0	0	0	0	6	0.15	0.45
Abortion-late	0	0	0	0	3	0.02	0.16
	0	0	0	0	4	0.01	0.11
Summary-IUFD	0	0	0	0	2	0.01	0.11
	0	0	0	0	2	0	0.07
Summary-mole	0	0	0	0	1	0	0.06
	0	0	0	0	2	0	0.04
Summary-EP	0	0	0	0	1	0.01	0.11
	0	0	0	0	1	0.02	0.12
Summary-Preterm	0	0	0	0	3	0.03	0.2
	0	0	0	0	5	0.02	0.16
Summary-term	0	0	0	0	2	0.06	0.25
	0	0	0	0	4	0.06	0.26
Summary-living child	0	0	0	0	2	0.06	0.24
	0	0	0	0	4	0.06	0.25
IUI Hx-total	2	2	2	3	10	2.73	1.47
	2	2	2	3	11	2.7	1.4
Result-failed	0	0	0	0.41	6	0.39	0.78
	0	0	0	1	10	0.41	0.78
Result-pregnant	0	0	0	0	2	0.04	0.2
	0	0	0	0	2	0.02	0.14
Summary of ART cycle-IVF	0	0	0	0	2	0.01	0.13
	0	0	0	0	6	0.03	0.24
Summary of ART cycle-ZIFT	0	0	0	0	1	0	0.02
	0	0	0	0	3	0	0.06
Summary of ART cycle-E.T freeze	1	1	1	1	4	1.01	0.11
	1	1	1	1	5	1.01	0.1
Summary of ART cycle-Total	0	0	0	0	4	0.02	0.2
	0	0	0	0	9	0.04	0.31
Physical exam-height	142	158	161.05	165	187	161.61	5.74
	142	157	161.05	164	187	160.96	5.7
Physical exam-weight	40	61	67.31	74	170	68.14	12.54
	36	60	67	73	171	67.18	11.65
Physical exam-BMI	15.02	23.53	25.93	28.04	52.11	26.01	4.23
	14.19	23.34	25.93	28.01	57.09	25.92	4.22
IUI clinic-number of cycle	1	1	1	2	5	1.35	0.63
	1	1	1	2	7	1.31	0.59
Infertility medical treatment-Starting day of Stimulation (menstrual day)	1	3	3	3.42	8	3.39	0.73
	1	3	3	3.42	14	3.42	0.82
Letrosol dose (mg/day)	0	0	0	1.12	7.5	1.28	2.04
	0	0	0	1.12	7.5	1.1	1.9
Letrosol duration	0	0	0	1.19	12	1.34	2.17
	0	0	0	1.19	15	1.16	2.05
Letrosol start day of stimulation	0	0	0	0.77	5	0.86	1.41
	0	0	0	0.77	17	0.76	1.38
Clomiphen dose (mg/day)	0	0	59.87	100	150	57.97	45.82
	0	0	100	100	150	60.19	45.54
Clomiphen duration	0	0	3	5	12	2.94	2.33
	0	0	5	5	15	3.07	2.36
Clomiphen Start day of stimulation	0	0	2.08	3	6	2	1.66
	0	0	2.08	3	18	2.09	1.7
Summary of foliculogenesis-duration of stimulation	1	9	10.23	11	25	10.41	2.59
	1	9	10.23	11	31	10.2	2.63
Fostimon dose IU	0	0	0	77.03	337.5	91.4	23.39
	0	0	0	77.03	247.5	74.61	18.54
Gonal.F dose IU	0	0	0	0	3750	60.96	26.71
	0	0	0	0	6847	60.2	23.93
Menopour Dose IU	0	0	0	0	2700	10.65	125.74
	0	0	0	0	3300	9.74	104.92
HMG dose IU	0	0	0	150	2625	116.59	221.67
	0	0	0	150	3300	125.45	230.94
Merional dose IU	0	0	0	0	3900	66.25	295.78
	0	0	0	0	9075	44.76	202.57
Hemogon dose IU	0	0	0	0	1200	3.09	44.01
	0	0	0	0	3000	4.95	55.65
Menogan dose IU	0	0	0	0	3450	46.63	195.23
	0	0	0	0	3600	44.2	163.25
Bravelle dose IU	0	0	0	0	1050	7.93	57.94
	0	0	0	0	2100	9.55	77.96
Gonadotropin total dose	0	225	375	450	4500	453.57	502.6
	0	225	375	450	4424	411.52	410.99
Esteradiol dose	0	0	0	0	6	0.33	0.99
	0	0	0	0	8	0.42	1.16
Esteradiol duration	0	0	0	0	15	0.25	0.95
	0	0	0	0	14	0.3	0.96
No of dominate follicle at HCG day_17	0	1	1.65	2	10	1.81	1.46
	0	1	1.65	2	11	1.61	1.38
No of dominate follicle at HCG day_18	0	0	0.95	1	7	0.97	0.95
	0	0	0.94	1	9	0.92	0.87
HCG day endometrium (endometrial thickness)	4.3	8	8.51	9	19	8.67	1.47
	1	8	8.52	9	18	8.48	1.51
Age_y	20	30	33	35	72	32.82	4.7
	20	30	33	36	80	33.35	4.88
Family demographic information (Education_y)	1	2	3	4	7	3.38	1.23
	1	2	3	4	7	3.31	1.25
Specimen characteristics-abstinence	0.41	3	4	4.59	20	4.42	1.79
	0.41	3	4	4.59	20	4.43	1.89
Specimen characteristics-volume (normal range 2.7 mL)	0.1	2	3.2	4	9.5	3.29	1.56
	0.1	2	3	4	10.5	3.23	1.54
Specimen characteristics-PH	6	7.8	7.8	7.8	8.5	7.8	0.11
	6	7.8	7.8	7.8	85	7.8	0.10
Specimen characteristics liquefaction time (normal range15-30 min)	20	20	20	30	60	26.10	8.60
	15	20	20	25.53	60	25.34	8.48
Sperm concentration- total sperm count	10	104	170.1	202.72	399	165.46	84.57
	10	92.5	168	204	400	163.60	88.18
Sperm motility-total motility (normal range 50)	10	40	57.3	72.22	98.8	55.94	19.87
	10	37.6	55.9	72.22	100	54.51	21.10
Total motile sperm count	1.14	41.06	86.70	132.90	354.88	95.29	64.21
	1	35.52	81.55	132.90	370.11	93.71	69.08
Sperm motility-shaking grade	0	0	0	0	5	0.03	0.36
	0	0	0	0	15	0.03	0.48
Sperm motility-grade I-	0	0	0	0	19	0.12	1.14
	0	0	0	0	34.3	0.12	1.16
Sperm motility-grade I (grade d)	0	0	3.6	8.2	37.3	5.3	5.92
	0	0	3.6	8.2	46.4	5.40	6.18
Sperm motility Grade II-	0	0	0	0	50.3	0.27	3.04
	0	0	0	0	57.6	0.21	2.57
Sperm motility Grade II (grade c)	0	10.27	26.7	36.8	64.7	24.11	16.26
	0	11.9	26.4	36.7	70.0	24.25	15.97
Sperm motility Grade II +	0	0	0	0	23.2	0.01	0.58
	0	0	0	0	37.3	0	0.38
Sperm motility Grade III (a + b)	0	2.7	9.8	18.6	48.9	11.96	10.76
	0	3.3	9.8	17.8	49.8	11.96	10.82
Sperm morphology-normal morphology (normal range 30)	0	4	5.62	7	20	5.72	2.99
	0	4	5.62	7	28	5.6	3.1
Sperm morphology-abnormal morphology (normal range 70)	80	93	94.38	96	100	94.27	2.99
	72	93	94.38	96	133	94.4	3.13
Sperm morphology-amorph	0	69	76	80	92	68.59	22.61
	0	68.66	75	80	96	68.68	21.7
Sperm morphology-double head	0	0	0	1.02	8	0.85	1.20
	0	0	1	2	11	1.02	1.36
Sperm morphology-giant head	0	1	2	4	20	2.40	2.51
	0	1	2	4	25	2.53	2.61
Sperm morphology-pin head	0	1	2	4	30	2.95	3.47
	0	1	2	4	30	3.14	3.37
Sperm morphology-round head	0	1	2	4	20	2.85	2.92
	0	1	2	4	20	2.66	2.69
Sperm morphology-double tail	0	0	0	0	4	0.14	0.47
	0	0	0	0	10	0.19	0.63
Sperm morphology-coiled tail	0	1	3	5	25	3.79	3.48
	0	2	3	6	28	4.17	3.84
Sperm morphology-short tail	0	1	1	2.31	24	1.90	2.37
	0	1	1	3	28	2.20	2.75
Sperm morphology-cytoplasmic droplet	1	4	7	14	20	8.08	4.94
	1	4	7	12	30	8.12	4.85
Other tests-germinal cell	0	1	2	3	15	2.14	1.87
	0	1	2	3	21	2.20	1.79
Therapeutic duration of liquefaction (min)	5	15	16.13	16.13	145	16.16	5.70
	5	15	16.13	16.13	200	16.15	5.22
Therapeutic volume	0.5	2.5	3.59	4	13	3.6	1.64
	0.01	2.5	3.5	4	13	3.54	1.66
Therapeutic PH	5	7.76	7.8	7.8	7.9	7.77	0.09
	0.33	7.76	7.8	7.8	10	7.76	0.19
Before processing motility	0.04	0.3	0.37	0.45	0.92	0.38	0.14
	0.01	0.28	0.37	0.45	0.98	0.37	0.14
Before processing sperm concentration	4	45	54.62	68	110	55.57	18.7
	1	42	54.62	68	110	54.15	20.23
After processing motility	0.2	0.96	0.98	1	1	0.97	0.06
	0.02	0.96	0.98	1	1	0.96	0.09
Post wash total motile sperm count	1	127.4	209.88	264.87	460	213.81	27.49
	0.16	102.9	195	252	450	198.81	35.09
After processing sperm concentration	2	50	60.7	72	120	60.82	21.94
	1	42	60.7	72	120	57.23	24.87

^*^Statistical description of numeric variables: Each row is divided into two parts in which the top row is related to the positive class and the bottom row represents the negative class

## Abbreviations

ACECR: Academic center for education, culture and research
AMH: Anti-müllerian hormone
ANN: Artificial neural networks
ART: Assisted reproductive technology
AUC: Area under curve
BMI: Body mass index
CN1: Complex network which is comprised of all the training data records as its nodes
CN2: Complex network which includes all training data excluding negative class
CN3: Complex network which includes all training data excluding positive class
CNFE-SE: Complex network-based feature engineering and stacked ensemble
C.V.: Cross validation
DSS: Decision support system
DT: Decision tree
FN: False negative
FP: False Positive
FSH: Follicle-stimulating hormone
HCA: Hierarchical clustering analysis
ICSI: Intracytoplasmic injection
IUI: Intrauterine Insemination
IVF: In-vitro fertilization (IVF)
K-NN: K-nearest neighbors
LH: Luteinizing Hormone
LR: Logistic regression
MDA: Mean decrease of accuracy
MLP: Multi-layer perceptron
N: Negative
NB: Naïve Bayes
P: Positive
PCA: Principal component analysis
RF: Random forest
RBF: Radial basis function
SVM: Support vector machines
SD: Standard deviation
TN: True negative
TP: True positive
TSH: Thyroid-stimulating hormone
